# Altering the balance between AOX1A and NDB2 expression affects a common set of transcripts in Arabidopsis

**DOI:** 10.3389/fpls.2022.876843

**Published:** 2022-11-15

**Authors:** Crystal Sweetman, Christopher D. Waterman, Darren C.J. Wong, David A. Day, Colin L.D. Jenkins, Kathleen L. Soole

**Affiliations:** ^1^ College of Science & Engineering, Flinders University, Bedford Park, SA, Australia; ^2^ College of Science, Australian National University, Canberra, ACT, Australia

**Keywords:** plant mitochondria, alternative oxidase, type II NAD(P)H dehydrogenase, overexpression, knockout, transcriptome, balancing act

## Abstract

Stress-responsive components of the mitochondrial alternative electron transport pathway have the capacity to improve tolerance of plants to abiotic stress, particularly the alternative oxidase AOX1A but also external NAD(P)H dehydrogenases such as NDB2, in Arabidopsis. NDB2 and AOX1A can cooperate to entirely circumvent the classical electron transport chain in Arabidopsis mitochondria. Overexpression of AOX1A or NDB2 alone can have slightly negative impacts on plant growth under optimal conditions, while simultaneous overexpression of NDB2 and AOX1A can reverse these phenotypic effects. We have taken a global transcriptomic approach to better understand the molecular shifts that occur due to overexpression of AOX1A alone and with concomitant overexpression of NDB2. Of the transcripts that were significantly up- or down- regulated in the AOX1A overexpression line compared to wild type (410 and 408, respectively), the majority (372 and 337, respectively) reverted to wild type levels in the dual overexpression line. Several mechanisms for the AOX1A overexpression phenotype are proposed based on the functional classification of these 709 genes, which can be used to guide future experiments. Only 28 genes were uniquely up- or down-regulated when NDB2 was overexpressed in the AOX1A overexpression line. On the other hand, many unique genes were deregulated in the NDB2 knockout line. Furthermore, several changes in transcript abundance seen in the NDB2 knockout line were consistent with changes in the AOX1A overexpression line. The results suggest that an imbalance in AOX1A:NDB2 protein levels caused by under- or over-expression of either component, triggers a common set of transcriptional responses that may be important in mitochondrial redox regulation. The most significant changes were transcripts associated with photosynthesis, secondary metabolism and oxidative stress responses.

## 1 Introduction

The importance of alternative oxidase (AOX) in mitigating oxidative stress is well documented and the mechanisms underlying this are popular research topics (Maxwell et al., 1999; Giraud et al., 2008; Watanabe et al., 2008; Florez-Sarasa et al., 2011; Panda et al., 2013; Vishwakarma et al., 2015; Del-Saz et al., 2016; Demircan et al., 2020; Jayawardhane et al., 2020). Transgenic *Arabidopsis thaliana* lines with modified expression of AOX1A, generated in the laboratory of Jim Siedow (Fiorani et al., 2005; Umbach et al., 2005), together with similar transgenic tobacco lines (Amirsadeghi et al., 2006) have been a cornerstone for understanding the roles of AOX (Smith et al., 2009; Skirycz et al., 2010; Wang et al., 2011; Cvetkovska and Vanlerberghe, 2012; Dahal et al., 2014; Liu et al., 2014; Dahal et al., 2015; Dahal and Vanlerberghe, 2017). These and other studies have confirmed the role of AOX in minimising the production of reactive oxygen species (ROS) in mitochondria by preventing over-reduction of the ubiquinone pool (for a review, see Vanlerberghe, 2013). Studies with AOX knockdown lines have noted an extra strain on photosynthesis and growth when these plants are exposed to stress (Giraud et al., 2008; Yoshida et al., 2011; Gandin et al., 2012; Dahal et al., 2014; Jiang et al., 2019), while AOX overexpression can protect photosynthetic machinery by utilising excess reducing equivalents and preventing ROS accumulation in the chloroplast (Dahal et al., 2014; Dahal et al., 2015; Dahal and Vanlerberghe, 2017). AOX isoforms have even been shown to functionally replace plastid localised PTOX in the *immutans* variegation mutant (Fu et al., 2012). Interrupting AOX expression affects the transcription of extramitochondrial proteins (Umbach et al., 2005; Clifton et al., 2006) and evidence for an intricate network of mitochondrial and chloroplast retrograde signaling factors is emerging (for a recent review, see Wang et al., 2020).

Relatively few studies have assessed the effects of modifying the expression of type II NAD(P)H dehydrogenases (NDs) on plant growth and stress tolerance, a task that is complicated by the dual targeting of NDAs, NDC, and NDB1 to other organelles in the cell as well as the mitochondrion (Carrie et al., 2008). Various *Arabidopsis thaliana* lines with knockdown or knockout of ND genes have now been reported for all except the non-expressed AtNDB3 (Smith et al., 2011; Wallström et al., 2014a; Wallström et al., 2014b; Fatihi et al., 2015; Sweetman et al., 2019). Simultaneous knockdown of both NDA proteins, or NDB1, by RNA interference resulted in delayed growth without affecting photosynthesis *per se* (Wallström et al., 2014a; Wallström et al., 2014b). Knockdown of AtNDB4 by RNA interference and TDNA insertion also resulted in growth delays during early development, however this disappeared during subsequent growth and the plants ultimately showed improved growth and resilience to salinity stress, attributed to the increase in endogenous NDB2 and AOX1a in these lines (Smith et al., 2011). Knockout of NDB2 by TDNA insertion had no obvious growth phenotype but caused increased sensitivity to a combined drought and high light stress (Sweetman et al., 2019). Knockout of NDC1 had no obvious effect on growth nor photosynthesis under optimal growth conditions (Piller et al., 2011), but growth and photosystem II efficiency were restricted upon exposure to high light (Fatihi et al., 2015). Interestingly, the latter studies also showed that NDC1 is a key enzyme for vitamin K1 biosynthesis.

The five *AOX* and seven *ND* isoforms of *A. thaliana* are expressed differently according to location, life cycle and external stimuli (Michalecka et al., 2003; Escobar et al., 2004; Clifton et al., 2006; Carrie et al., 2008). Each transcript has been identified as responsive to at least one stressor or treatment (Clifton et al., 2005; Elhafez et al., 2006; Feng et al., 2013) and some appear to be co-regulated in response to stress, particularly *AOX1A, NDA2* and *NDB2* (Clifton et al., 2005; Ho et al., 2008; Smith et al., 2009; Vijayraghavan and Soole, 2010). Physical association of some AOX and ND proteins has been observed in non-denaturing PAGE experiments (Senkler et al., 2017). Functional cooperation of AOX1A and NDB2 was also demonstrated recently in isolated mitochondria: over-expression of NDB2 alone resulted in high levels of largely inactive protein, which became active when co-expressed with AOX1A (Sweetman et al., 2019). Such cooperation between ND and AOX isoforms provides a complete bypass of the classical ETC. Measurements using these *A. thaliana* lines with modified AOX1A and NDB2 expression highlighted differences in their ability to grow under non-limiting conditions, and to survive and recover from a combined drought and increased light stress (Sweetman et al., 2019). Specifically, plants over-expressing AOX1A alone or AOX1A and NDB2 together, were more stress-resilient and capable of recovery than wild type plants, or plants overexpressing NDB2 alone, while plants lacking either AOX1A or NDB2 were severely impacted. However, the molecular mechanisms behind these phenotypes have yet to be determined. In addition, plants over-expressing AOX1A alone exhibited a small growth delay under standard growth conditions (not an isolated incident, see Skirycz et al., 2010) that was rectified by concomitant NDB2 overexpression.

In the present study, a global approach has been used to determine the effects of altered AOX1A and NDB2 proteins on growth-, metabolism- and stress-related gene transcripts in plants grown under standard conditions. The plant lines investigated included one of the original AOX1A-overexpression lines (AOX1A-OEX) generated in the Siedow laboratory (Umbach et al., 2005), an AOX1A and NDB2 dual overexpression line (dual-OEX) developed recently by overexpressing NDB2 in the AOX1A overexpression line (Sweetman et al., 2019), and an NDB2 knockout line (*ndb2*) from the same study. We address the following question: what mechanisms, driven by transcriptional changes, could cause the growth delay phenotype in plants over-expressing AOX1a alone, and how is this rectified by concomitant NDB2 overexpression? A second question arose during the analysis: namely, is there a common transcriptional response to alterations in the ratio of AOX1A:NDB2?

## 2 Methods

### 2.1 Plant materials and growth conditions

Four plant lines were used in this study; wild type (WT; *A. thaliana* Col0), an AOX1A overexpression line kindly provided by Jim Siedow’s laboratory many years ago (AOX1A-OEX; XX1; Umbach et al., 2005), an AOX1A and NDB2 dual overexpression line (dual-OEX; 5.2; Sweetman et al., 2019) and an NDB2 TDNA insertion line (*ndb2*; SALK_036330; Sweetman et al., 2019). The “single” NDB2 overexpression lines reported by Sweetman et al. (2019) were not analysed here, as enhanced NDB2 activity could not be detected in those lines (unless AOX1A was also overexpressed). *A. thaliana* plants were grown in a controlled temperature cabinet at 20°C with 16h day length and PAR of 80-120 μmols.m^-2^.sec^-1^ using custom-made panels of red and blue LED light modules (Phoenix Biosystems, Australia). A coco-peat soil mix (PIRSA-SARDI, Australia) was supplemented with slow-release fertiliser with trace elements (Osmocote, Australia) and used to fill ~6 cm diameter pots, each harbouring a single *A. thaliana* plant. Seedlings were germinated directly on soil, watered every 2-3 days and positions rotated regularly within the cabinet. Whole rosettes were harvested at 44 days after sowing, flash frozen in liquid nitrogen, pulverised using a mortar and pestle and stored at -80°C until use.

### 2.2 RNA extraction and sequencing

Total RNA was extracted from frozen tissue powder using the Isolate II RNA Plant kit (Bioline, NSW). RNA quality was assessed using the Perkin Elmer LabChip GX Touch 24 with the RNA quality score cut off set at >8. mRNA was purified and cDNA prepared using the Truseq Stranded mRNA HT Sample Prep Kit (Illumina).

Flinders Genomics Facility, Adelaide, Australia, provided RNA sequencing services. cDNA libraries were analysed with the Perkin Elmer LabChip GX Touch 24 using the High Sensitivity DNA kit producing an average fragment size of 268 bp. Libraries were sequenced using the Illumina NextSeq 500 to generate single-end reads of 75 bp. Raw sequence reads were then processed using the following pipeline: (1) remove reads with sequence quality scores less than Q30; (2) remove adapter/overrepresented sequence and cross species contamination; (3) remove reads with final lengths less than 30 bp. The quality of the cleaned reads was assessed using FastQC. The cleaned sequence reads were then aligned and mapped against the *Arabidopsis* genome (Ensembl, TAIR10) using hisat2 aligner (v2.2.1). Read counts were obtained using FeatureCounts (Liao et al., 2014) with default parameters except for –C (exclude chimeric fragments) option enabled. A summary of read data are included in **Table S1**.

DESeq2 was used for statistical analysis of differential expression. Pre-filtering of lowly-expressed genes was enabled prior to differential expression comparisons for each line relative to WT. Differentially Expressed Genes were defined as log2 fold changes greater than 1 or less than -1, with p-adjusted values <0.05. All associated expression (e.g. normalised counts, RPKM) and differential expression results tables (containing the baseMean, log2FoldChange and FDR) are included in **Table S2**.

### 2.3 Functional enrichment analysis

Assessment of enriched functional categories in up- and downregulated DEGs in various lines relative to the WT were performed using BiNGO, using the hypergeometric distribution-adjusted Benjamini & Hochberg false discovery rate (FDR) for multiple hypothesis correction (Maere et al., 2005). Plant Gene Ontology (GO) slim categories and in-depth biological process (BP) were evaluated and an FDR-adjusted p value < 0.05 indicates enriched GO functional categories for a given comparison. Enrichment of upstream TFs were identified using EAT-UpTF based off the relative enrichment of TF-target genes (Shim and Seo, 2020). A FDR-adjusted p value < 0.01 indicates significantly enriched TFs in up- and downregulated DEGs. All associated visualisation of enriched categories were performed with ggplot2 (https://ggplot2.tidyverse.org/). Commonalities and differences of up- and downregulated DEGs between various lines were identified using Jvenn (Bardou et al., 2014). Transcriptomic data (log2 FC, FDR-adjusted p-value <0.05) were visualized using MAPMAN 3.0.0 (Thimm et al., 2004).

### 2.4 Malondialdehyde measurements

Lipid peroxidation levels were estimated using the spectrophotometric TBARS method for measuring malondialdehyde (MDA) equivalents, as described by Hodges et al. (1999) and Singh et al. (2012). Approximately 50 mg of frozen leaf powder was extracted in 1 ml of 80% (v/v) ethanol and centrifuged at 16,000 g for 15 minutes. The supernatant was divided into two aliquots. Each aliquot was mixed with an equal volume of assay mix (20% (w/v) trichloroacetic acid and 0.01% (w/v) B-hydroxybutyric acid) either with or without 0.01% (w/v) thiobarbituric acid. Samples were heated to 96°C for 30 minutes and then quickly cooled on ice. After centrifugation at 9,500 g for 10 minutes, absorbance was measured at 440, 532 and 600 nm, and MDA equivalents calculated according to Hodges et al. (1999).

## 3 Results and discussion

### 3.1 Transcriptomic effects of AOX1A and NDB2 manipulation

**Figure 1:**
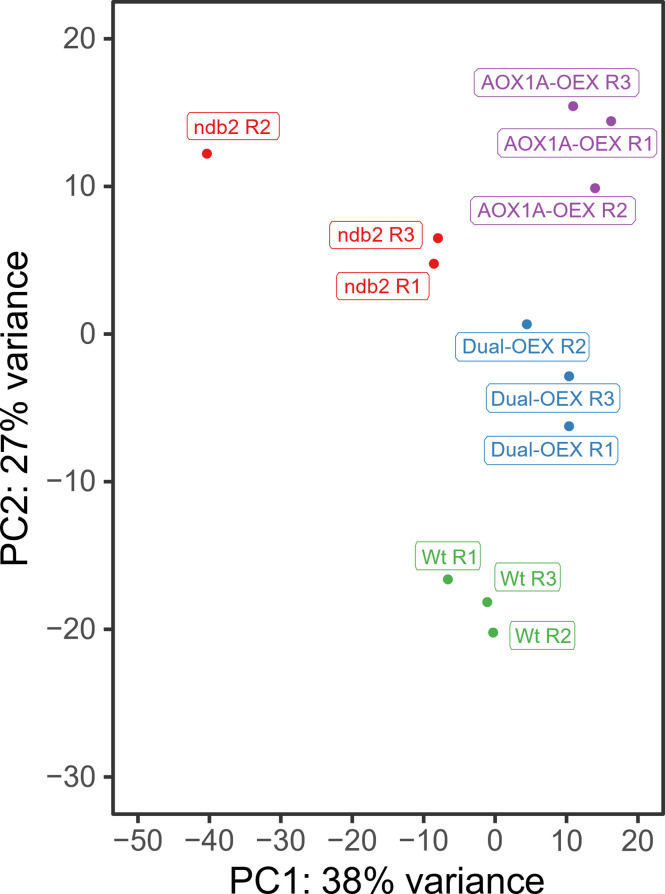
PCA plot of RNAseq data. Each point represents an individual replicate of wild type (Wt, green), AOX1A-OEX (purple), dual-OEX (blue) and *ndb2* (red). The proportion of variance explained by PC1 and PC2 are indicated in the axis titles.

Principal Component Analysis (PCA) (**Figure 1**) showed that the different lines were transcriptionally distinct but generally consistent between replicates, with PC1 and PC2 explaining 38 and 27% of total variation, respectively. Replicate 2 of *ndb2* showed separation from other replicates, but remained distinct from the other lines, and clear differences in transcript levels of *ndb2* compared to wild type were still observed. Clustering patterns suggested that (1) the transcriptome of AOX1A-OEX was very different from the transcriptome of WT plants; (2) concomitant AOX1A and NDB2 overexpression partially caused a reversion towards the WT transcriptome and (3) the *ndb2* transcriptome was different from all other lines on the PC1 axis, but similar to that of the AOX1A-OEX line on the PC2 axis. To further explore these relationships, differentially expressed genes (DEGs) were determined for the AOX1A-OEX, dual-OEX and *ndb2* lines relative to WT plants (|log2FC| > 1, FDR-adjusted p-value < 5%).

**Figure 2:**
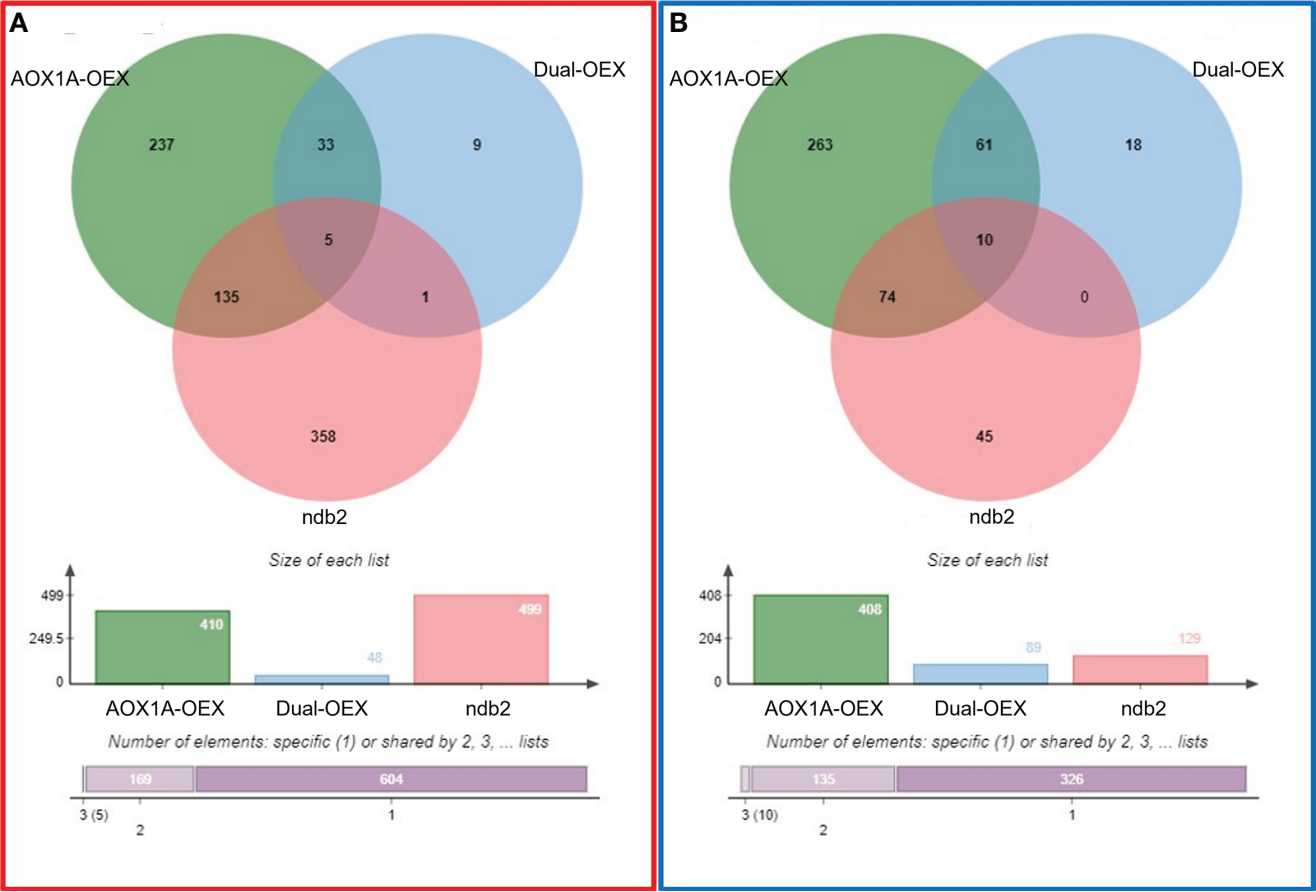
Commonalities and differences between **(A)** up-regulated and **(B)** down-regulated DEGs, relative to wild type. AOX1A-OEX (green), dual-OEX (blue) and *ndb2* (red).

To reduce “wasteful” respiratory flux, overexpressed AOX1A protein might be maintained in an inert state when not required, *via* post-translational controls. Nonetheless, a total of 818 (410 up- and 408 down-regulated) DEGs were observed in the AOX1A-OEX line (**Figure 2**). Therefore, it is likely that the excess protein is at least partially active and alters respiratory properties of the tissue. Conversely, overexpression of both AOX1A and NDB2 in the dual-OEX line led to fewer DEGs, with only 48 and 89 transcripts up- and down-regulated, respectively. That is, almost 90% of up-regulated gene transcripts and almost 80% of down-regulated transcripts in the AOX1A-OEX line were no longer differentially expressed in the dual-OEX line. Only 10 up-regulated and 18 down-regulated DEGs in the dual-OEX line were unique changes: all others were also found in the single AOX1A-OEX line. (**Figure 2**; **Table S3**). This suggests that overexpression of NDB2 together with AOX1A caused a major reversion of the AOX1A-OEX transcriptome, in agreement with the PCA, and overexpression of NDB2 in the AOX1A background led to only a few novel transcriptional changes. Of the latter, a nicotianamine synthase gene and an ABC transporter involved in auxin efflux were up-regulated, while an UMAMI transporter and a QQS gene involved in regulating carbon/nitrogen balance were down-regulated.

Of the 628 DEGs in the *ndb2* line, 80% (499 transcripts) were up-regulated and only 20% (129 transcripts) down-regulated. A surprising and potentially important finding was the level of commonality between DEGs in lines with alternative pathway genes knocked out (*ndb2*) and overexpressed (AOX1A-OEX): approximately 28% and 65% of transcripts were commonly up- and down-regulated in these lines (224 transcripts in total). However, there was also a large set of unique responses in both lines (**Figure 2**). The set of commonly up- and down-regulated transcripts featured genes involved in development and growth (e.g. ARGOS-like genes), photosynthesis (e.g. photosystem II reaction centre proteins), respiration (e.g. a succinate dehydrogenase subunit), lipid metabolism (e.g. oleosin genes), secondary metabolism (e.g. dihydroflavonol reductase), and abiotic and biotic stress responses (e.g. defensin and DREB genes). These are described in more detail in later sections.

The relationships between AOX1A-OEX, dual-OEX and *ndb2* were further explored through Gene Ontology profiling, MapMan visualisation and a survey of the most affected transcripts in each line.

### 3.2 Gene ontology enrichment profiles

**Figure 3:**
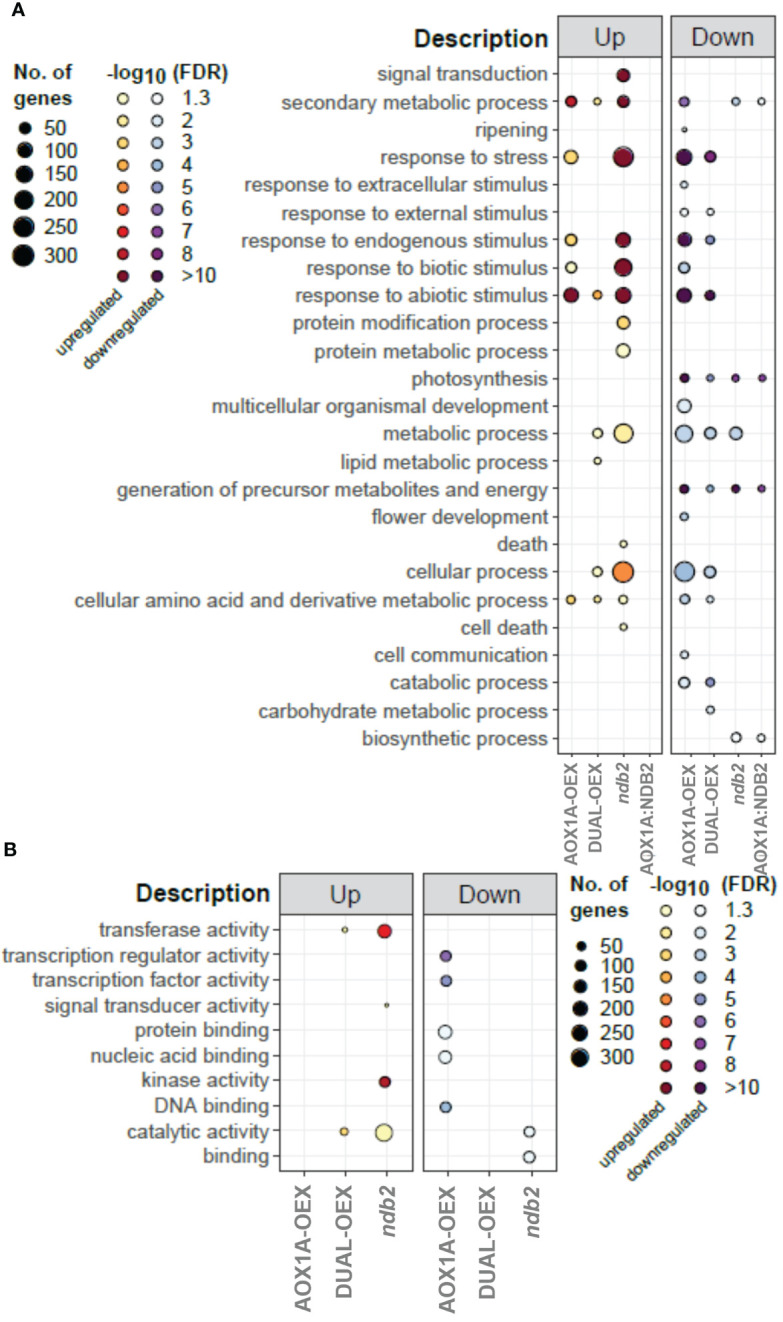
Enrichment of gene ontology terms between lines. BinGO was used to profile the enrichment of GO SLIM terms of individual lines, relative to wild type. The set of DEGs that are potentially regulated by AOX1A:NDB2 were also profiled separately, relative to wild type. Larger data points represent a larger number of genes within that category, while greater colour intensity represents greater significance (-log_10_FDR). **(A)** biological processes, **(B)** molecular functions.

Comparisons between transgenic lines enabled identification of ontologies that were significantly over-represented relative to WT. While there was a considerable level of overlap, particularly between AOX1A-OEX and *ndb2* lines, a number of ontologies were also represented uniquely in individual lines (**Figure 3**). Enrichment of “signal transduction” and “respone to stress” terms were seen in up-regulated transcripts of the *ndb2* line, relating to both biotic and abiotic stimuli. Cell death also featured in this line, as well as protein modification and metabolism and a significant enrichment of “metabolic process” and “cellular process”. On the other hand, the AOX1A-OEX line demonstrated a decrease in “cellular process”, while “response to stress” terms were enriched in both up- and down-regulated transcripts, particularly the latter, and could be mostly related to “response to abiotic stimulus”. The AOX1A-OEX line generally showed a stronger enrichment of ontologies among down-regulated transcripts compared to up-regulated transcripts, including terms related to development and stress. Enhanced AOX expression has been linked previously with resistance to several different abiotic stress conditions (Smith et al., 2009; Cvetkovska and Vanlerberghe, 2013; Dahal et al., 2015; Yang et al., 2019) and our results suggest that constitutive expression of AOX may prepare the plant for such stress responses. The down-regulation of development-related transcripts could relate to the delayed growth phenotype that was observed in this line but not in the other two lines (Sweetman et al., 2019).

The only unique enrichment terms seen for the dual-OEX line were an increase in lipid metabolic process and a decrease in carbohydrate metabolic process. Due to the lack of growth phenotype in this line (Sweetman et al., 2019), changes in transcript levels of the genes within these functional categories either do not affect protein activity, or if they do, the changes do not affect growth under non-limiting conditions. Knocking out NDB2, on the other hand, caused a large enrichment of genes involved in the response to stress and cell death, which, while not manifesting in growth changes under standard conditions, could have led to the drought and light stress sensitivity of this line (Sweetman et al., 2019).

### 3.3 The largest transcriptional responses to altered AOX1A and NDB2 expression

#### 3.3.1 The top DEGs of the AOX1A-OEX line

The top 20 up-regulated DEGs in the AOX1A-OEX line were expressed 9.5 to 50-fold higher than wild type transcript levels. None of these, except the *AOX1A* transgene, were up-regulated in the dual-OEX line. The largest transcript induction in the AOX1A-OEX line was for a defensin-like (DEFL) family protein gene (over 50-fold, **Table 1**), which suggested a strong defense response when AOX1A was over-expressed alone. Consistent with this, *MIR863A*, a microRNA thought to be involved in biotic defense (Niu et al., 2016), *UNUSUAL SERINE PROTEASE INHIBITOR (UPI)* and the POLYGALACTURONASE ABSCISSION ZONE A. THALIANA (PGAZAT) gene AD*PG1*, also featured within the top 20 most up-regulated transcripts. ADPG genes are thought to be involved in releasing pathogenesis-related signaling molecules from the plant cell wall (Ogawa et al., 2009; Gallego-Giraldo et al., 2020). AOX expression is induced also by biotic stress (Zhang et al., 2012) and constitutive over expression of AOX seems to prime the plant for increased stress resilience. Other top 20 up-regulated genes were: two small nucleolar RNAs (snoRNA); *ROP (RHO OF PLANTS) GUANINE NUCLEOTIDE EXCHANGE FACTOR 13 (ROPGEF13)*, which may be a GDP/GTP exchange protein for a Rho of plants signal switch (Berken et al., 2005; Shin et al., 2010); *1-DEOXY-D-XYLULOSE 5-PHOSPHATE SYNTHASE 1 (DXPS1)*, which belongs to a gene family involved in the non-mevalonate pathway of isprenoid biosynthesis (Lange and Ghassemian, 2003), and *AO4*, an aldehyde oxidase.

**Table 1:** Top 20 up-regulated DEGs for AOX1A overexpression line relative to wild type.

Gene Locus	Gene Name/Description	AOX1A-OEX		Dual-OEX		*ndb2*	
		FC	FDR	FC	FDR	FC	FDR
AT1G13609	defensin-like (DEFL)	52.34	**4.1E-05**	7.55	1.0E+00	11.44	**2.4E-02**
AT3G22370	AOX1A	39.61	**3.6E-91**	35.39	**1.4E-85**	1.38	1.9E-01
AT5G06165	other_RNA	34.37	**5.5E-04**	4.64	1.0E+00	31.77	**1.1E-03**
AT1G65541	hypothetical protein	31.41	**1.3E-03**	9.13	1.0E+00	28.70	**2.6E-03**
AT3G05932	Natural antisense transcript	23.36	**1.9E-03**	6.30	1.0E+00	26.36	**1.6E-03**
AT3G49551	unknown protein	21.74	**4.1E-03**	2.37	1.0E+00	11.65	**3.5E-02**
AT2G23755	transmembrane 220 helix protein	19.18	**7.6E-04**	4.23	1.0E+00	18.13	**1.4E-03**
AT4G13494	MIR863A	18.22	**9.4E-03**	3.11	1.0E+00	23.83	**4.9E-03**
AT3G47342	snoRNA	17.80	**5.9E-03**	5.55	1.0E+00	21.11	**4.2E-03**
AT3G16130	ROPGEF13	17.73	**5.0E-03**	8.73	1.0E+00	19.34	**4.8E-03**
AT3G21500	DXPS1	14.57	**1.1E-03**	2.20	1.0E+00	1.73	6.4E-01
AT2G14247	Expressed protein	13.60	**1.3E-03**	4.55	1.0E+00	7.52	**2.1E-02**
AT3G57510	ADPG1	11.54	**4.4E-02**	2.04	1.0E+00	0.58	7.8E-01
AT2G24592	hypothetical protein	10.72	**2.9E-06**	1.28	1.0E+00	9.98	**1.3E-05**
AT1G29357	Natural antisense transcript	10.69	**1.1E-02**	5.10	1.0E+00	7.93	**3.7E-02**
AT2G20722	snoRNA	10.52	**4.4E-02**	0.44	1.0E+00	9.55	6.4E-02
AT3G61898	transmembrane protein	10.19	**5.5E-05**	1.19	1.0E+00	5.99	**4.1E-03**
AT5G43580	UPI	10.01	**3.5E-03**	1.74	1.0E+00	0.92	9.6E-01
AT1G29418	transmembrane protein	9.66	**4.3E-11**	0.92	1.0E+00	8.80	9.0E-10
AT1G04580	AO4	9.54	**3.2E-03**	3.71	1.0E+00	1.30	8.3E-01
AT1G53542	hypothetical protein	9.51	**4.3E-06**	1.34	1.0E+00	4.76	**3.9E-03**

Including fold change values for each gene and each line for comparison. Statistical significance (FDR-adjusted p value < 5%) indicated in bold. AOX1A-OEX: AOX1A overexpression line, Dual-OEX: AOX1A and NDB2 overexpression line, *ndb2*: NDB2 knockout line.

The most strongly down-regulated transcript in the AOX1A-OEX line was *GLYCINE RICH PROTEIN 17 (GRP17)*, expressed at 0.5% of wild type levels (**Table 2**). This gene encodes an oleosin, important for maintaining the structure of oil bodies during seed dessication. In fact, six of the eight most strongly down-regulated genes (all transcribed to less than 2% of wild type levels) are involved in lipid transfer, seed storage, oil bodies and dessication, including another oleosin gene, *GRP19*. Both *GRP17* and *GRP19* are expressed during floral development and the corresponding proteins are found on mature pollen coats (Mayfield et al., 2001), although evidently the transcripts can also accumulate in the rosettes. Other genes that potentially relate to seeds (and all transcribed to less than 6% of wild type levels), included an esterase/acyltransferase/lipase that may be involved in reactions with lipids; an acyl-CoA synthetase that is active against medium- to long-chain fatty acids and involved in pollen formation; a tapetum-specific O-methyltransferase that may be involved with seed development and; *DOG1*, a QTL that is involved in controlling seed dormancy and is typically expressed in seed only. A class III peroxidase and *SEPALLATA3*, a MADS box transcription factor, both involved in floral development, were also strongly down-regulated to 3% and 5% of wild type levels, respectively. It is unclear why so many genes involved in flower and seed processes were expressed in the rosettes of Arabidopsis, and further, why they were down-regulated so strongly in the AOX1A-OEX line. Down-regulation of mitochondrial ROS might be involved, as ROS play integral roles in vegetative and reproductive development (see recent review by Huang et al., 2019). We have observed that seeds of the AOX-OEX lines tended to be smaller than those from WT plants (C. Sweetman unpublished results) and this may partially explain the growth delay seen in this line.

**Table 2:** Top 20 down-regulated DEGs for AOX1A overexpression line relative to wild type.

Gene Locus	Gene Name/Description	AOX1A-OEX		Dual-OEX		*ndb2*	
		FC	FDR	FC	FDR	FC	FDR
AT5G07530	GRP17	0.00	**2.2E-04**	0.01	1.0E+00	0.00	**3.1E-04**
AT3G05170	Phosphoglycerate mutase family	0.01	**8.4E-08**	0.01	1.0E+00	1.09	7.7E-01
AT1G66850	inhibitor/seed storage/LTP family protein	0.01	**1.8E-02**	0.11	1.0E+00	0.01	**2.1E-02**
AT3G51590	LTP12	0.01	**7.7E-04**	0.23	1.0E+00	0.19	1.9E-01
AT5G07550	GRP19	0.01	**1.3E-03**	0.12	1.0E+00	0.03	**1.8E-02**
AT1G68875	hypothetical protein	0.01	**2.9E-03**	0.30	1.0E+00	0.03	**1.6E-02**
AT1G47980	desiccation-like protein	0.02	**1.4E-03**	0.29	1.0E+00	1.04	9.8E-01
AT4G28395	ANTHER 7 (A7)	0.02	**2.6E-03**	0.92	1.0E+00	0.43	5.3E-01
AT1G75940	ATA27	0.02	**6.1E-03**	0.52	1.0E+00	0.09	9.1E-02
AT1G20150	Subtilisin-like serine endopeptidase family protein	0.02	**5.0E-03**	0.23	1.0E+00	0.42	5.4E-01
AT5G13380	Auxin-responsive GH3 family protein	0.02	**9.6E-03**	0.47	1.0E+00	0.36	5.2E-01
AT1G20130	GDSL-motif esterase/acyltransferase/lipase	0.03	**2.4E-03**	0.29	1.0E+00	0.07	**1.2E-02**
AT1G44970	PRX9	0.03	**3.4E-03**	1.74	1.0E+00	0.71	7.7E-01
AT1G62940	ACOS5	0.04	**1.7E-02**	0.47	1.0E+00	0.42	4.5E-01
AT1G11080	SCPL31	0.04	**1.2E-04**	0.52	1.0E+00	0.21	**2.2E-02**
AT2G01020	5.8SrRNA	0.05	**5.2E-15**	0.29	1.0E+00	0.13	**9.0E-12**
AT1G24260	SEP3	0.05	**1.8E-04**	0.81	1.0E+00	0.42	1.2E-01
AT1G67990	TSM1	0.05	**1.9E-02**	0.87	1.0E+00	0.31	3.1E-01
AT5G45830	DOG1	0.06	**1.1E-02**	0.29	1.0E+00	1.33	7.6E-01
AT3G63095	inhibitor/seed storage/LTP family protein	0.06	**1.1E-02**	0.63	1.0E+00	0.24	1.2E-01

Including fold change values for each gene and each line for comparison. Statistical significance (FDR < 5%) indicated in bold. AOX1A-OEX: AOX1A overexpression line, Dual-OEX: AOX1A and NDB2 overexpression line, *ndb2*: NDB2 knockout line.

#### 3.3.2 The top DEGs of the NDB2 knockout line

The *ndb2* line showed a small and non-significant reduction in *NDB2* transcript. This contradicts our previously described qPCR results (Sweetman et al., 2019) but can be explained by the RNAseq detection of a truncated mRNA. The first seven exons form a truncated transcript that cannot produce functional enzyme (**Figure S1**), while the qPCR assay targeted a region across the tenth and eleventh exons (Sweetman et al., 2019).

The *ndb2* transcriptome showed similarities to the AOX1A-OEX transcriptome, with 9 of the top 20 up-regulated genes in the *ndb2* line also up-regulated in the AOX1A-OEX line, along with 15 of the top 20 down-regulated genes (**Table 3**). This is consistent with the PCA clustering patterns and considerable overlap in Gene Ontology terms between lines. The most highly up-regulated genes unique to *ndb2* were largely involved in signal reception, including *RECEPTOR LIKE PROTEIN 11 (RLP11)*; *CYSTEINE-RICH RECEPTOR-LIKE PROTEIN KINASE 39 (CRK39)* and *37 (CRK37)*; and *CRINKLY4 RELATED 4 (CCR4)*, which were up-regulated between 16-60-fold. An unknown calmodulin-binding protein and transmembrane protein were also uniquely up-regulated in *ndb2*, along with two transcription factors, *NAC DOMAIN CONTAINING PROTEIN 90 (NAC090)* and *WRKY DNA-BINDING PROTEIN 30 (WRKY30)*, plus *UDP-GLUCOSYL TRANSFERASE 73D1 (UGT73D1)* and a heat shock protein.

**Table 3:** Top 20 up-regulated DEGs for NDB2 knockout line relative to wild type.

Gene Locus	Gene Name/Description	AOX1A-OEX		Dual-OEX		*ndb2*	
		FC	FDR	FC	FDR	FC	FDR
AT1G71390	RLP11	5.54	2.1E-01	3.16	1.0E+00	67.19	**3.1E-04**
AT5G06165	other_RNA	34.37	**5.5E-04**	4.64	1.0E+00	31.77	**1.1E-03**
AT4G04540	CRK39	2.78	5.2E-01	1.61	1.0E+00	30.26	**5.5E-03**
AT1G65541	hypothetical protein	31.41	**1.3E-03**	9.13	1.0E+00	28.70	**2.6E-03**
AT3G53150	UGT73D1	2.63	5.3E-01	2.51	1.0E+00	26.93	**7.7E-03**
AT3G05932	Potential natural antisense gene	23.36	**1.9E-03**	6.30	1.0E+00	26.36	**1.6E-03**
AT4G13494	MIR863A	18.22	**9.4E-03**	3.11	1.0E+00	23.83	**4.9E-03**
AT3G47342	snoRNA	17.80	**5.9E-03**	5.55	1.0E+00	21.11	**4.2E-03**
AT3G16130	ROPGEF13	17.73	**5.0E-03**	8.73	1.0E+00	19.34	**4.8E-03**
AT2G23755	transmembrane family 220 helix protein	19.18	**7.6E-04**	4.23	1.0E+00	18.13	**1.4E-03**
AT5G47850	CCR4	2.00	5.7E-01	4.06	1.0E+00	16.70	**2.3E-03**
AT1G09080	BIP3	2.08	4.2E-01	2.51	4.4E-01	15.73	**2.3E-04**
AT5G22380	NAC090	0.31	2.3E-01	1.20	1.0E+00	15.41	**4.9E-04**
AT1G53625	hypothetical protein	1.81	5.9E-01	1.07	1.0E+00	14.88	**9.4E-04**
AT5G57010	calmodulin-binding family protein	0.59	5.9E-01	1.13	1.0E+00	14.05	**3.9E-04**
AT4G02005	None	7.60	**2.1E-06**	1.33	1.0E+00	12.40	**3.1E-09**
AT5G24110	WRKY30	1.00	1.0E+00	1.25	1.0E+00	12.37	**1.9E-06**
AT4G04500	CRK37	3.34	8.0E-02	4.17	1.0E+00	12.34	**6.5E-05**
AT3G29000	Calcium-binding EF-hand family protein	4.19	**1.8E-02**	2.08	1.0E+00	11.80	**1.4E-05**
AT2G04495	transmembrane protein	2.28	1.8E-01	1.76	1.0E+00	11.69	**3.7E-06**

Including fold change values for each gene and each line for comparison. Statistical significance (FDR < 5%) indicated in bold. AOX1A-OEX: AOX1A overexpression line, Dual-OEX: AOX1A and NDB2 overexpression line, *ndb2*: NDB2 knockout line.

Transcripts that were uniquely down-regulated in the *ndb2* line included: *MADS AFFECTING FLOWERING 5 (MAF5)* and *4 (MAF4)*, which regulate flowering time, *WRKY DNA-BINDING PROTEIN 14 (WRKY14)*, *CYSTEINE ENDOPEPTIDASE 1 (CEP1)*, which is likely involved in cell death, vacuolar rupture and/or cell wall thickening (Han et al., 2019), and *SENESCENCE-ASSOCIATED GENE 29 (SAG29)*, which encodes a SWEET sucrose efflux transporter family protein (**Table 4**). All of these transcripts were down-regulated to less than 18% of wild type values and may be involved in regulating growth and development. Other down-regulated transcripts included an oil storage protein *FLORAL TRANSITION AT THE MERISTEM1 (FTM1)*, two 5.8SrRNA-like genes and two genes involved in flavonoid biosynthesis, *DIHYDROFLAVONOL 4-REDUCTASE (DFR)* and *LEUCOANTHOCYANIDIN DIOXYGENASE (LDOX)*.

**Table 4:** Top 20 down-regulated DEGs for NDB2 knockout line relative to wild type.

Gene Locus	Gene Name/Description	AOX1A-OEX			Dual-OEX			*ndb2*		
		FC	FDR	FC		FDR	FC		FDR
AT5G07530	GRP17	0.00	**2.2E-04**	0.01		1.0E+00	0.00		**3.1E-04**
AT1G66850	inhibitor/seed storage/LTP superfamily protein	0.01	**1.8E-02**	0.11		1.0E+00	0.01		**2.1E-02**
AT1G68875	hypothetical protein	0.01	**2.9E-03**	0.30		1.0E+00	0.03		**1.6E-02**
AT5G07550	GRP19	0.01	**1.3E-03**	0.12		1.0E+00	0.03		**1.8E-02**
AT5G65080	MAF5	0.26	1.0E-01	0.16		6.1E-02	0.04		**3.7E-05**
AT1G20130	GDSL-motif esterase/acyltransferase/lipase	0.03	**2.4E-03**	0.29		1.0E+00	0.07		**1.2E-02**
AT2G07698	ATPase, F1 complex, alpha subunit protein	0.15	**4.0E-03**	0.57		1.0E+00	0.09		**1.9E-03**
AT1G43800	FTM1	0.35	**4.0E-02**	0.14		1.0E+00	0.11		**2.5E-05**
AT2G05914	Potential natural antisense gene	0.15	**3.5E-02**	0.21		1.0E+00	0.12		**2.4E-02**
AT2G01020	5.8SrRNA	0.05	**5.2E-15**	0.29		1.0E+00	0.13		**9.0E-12**
AT1G23110	fold protein	0.36	2.1E-01	0.88		1.0E+00	0.14		**1.8E-02**
AT4G22880	LDOX	0.30	**3.1E-03**	0.39		8.4E-02	0.15		**6.3E-06**
AT1G30650	WRKY14	0.60	6.0E-01	0.72		1.0E+00	0.16		**3.7E-02**
AT5G50260	CEP1	0.94	9.4E-01	1.09		1.0E+00	0.16		**2.9E-02**
AT2G21260	NAD(P)-linked oxidoreductase superfamily protein	0.27	**4.7E-02**	0.21		1.0E+00	0.16		**1.1E-02**
AT2G15400	NRPE3B	0.29	**3.8E-03**	0.45		1.0E+00	0.17		**1.0E-04**
AT3G41979	5.8SrRNA	0.33	**3.3E-02**	2.52		1.0E+00	0.17		**1.1E-03**
AT5G42800	DFR	0.27	**1.5E-03**	0.27		**6.5E-03**	0.17		**2.1E-05**
AT5G13170	SAG29	0.72	7.1E-01	0.07		1.0E+00	0.17		**3.0E-02**
AT5G65070	MAF4	0.64	3.4E-01	0.45		1.0E+00	0.18		**4.0E-04**

Including fold change values for each gene and each line for comparison. Statistical significance (FDR < 5%) indicated in bold. AOX1A-OEX: AOX1A overexpression line, Dual-OEX: AOX1A and NDB2 overexpression line, *ndb2*: NDB2 knockout line.

#### 3.3.3 The top DEGs of the dual-OEX line

The vast majority of top-20 DEGs in the dual-OEX line were also differentially expressed in the AOX1A-OEX line. Only three were uniquely up-regulated in the dual-OEX line: the *NDB2* transgene, over 20-fold higher than wild type levels, *ProT3*, a proline transporter gene that was up-regulated 3.5-fold but was also very close to being significantly up-regulated in the AOX1A-OEX line, and *NAS3*, a nicotianamine synthase gene that was up-regulated 2.5-fold (**Table 5**). Interestingly, both *NDB2* and *NAS3* were up-regulated in response to UV-B treatments and this up-regulation was lost in *cop1* and *hy5* knockout plants, suggesting a COP1/HY5-mediated mechanism of transcription for *NDB2* and *NAS3* (Oravecz et al., 2006). COP1 is a positive regulator of transcriptional responses to low levels of UV-B and it acts upstream of HY5, which is involved in photomorphogenic development (Cluis et al., 2004). Neither *COP1* nor *HY5* were differentially expressed in the dual-OEX line, but a homolog of *HY5*, *HYH*, was up-regulated almost 4-fold. The possibility of COP1/HY5 acting as a regulator of *NDB2* transcription in response to changes in light quantity and quality warrants further investigation.

**Table 5:** Top 20 up-regulated DEGs for dual AOX1A and NDB2 overexpression line relative to wild type.

Gene Locus	Gene Name/Description	AOX1A-OEX		Dual-OEX		*ndb2*	
		FC	FDR	FC	FDR	FC	FDR
AT3G22370	AOX1A	39.61	**3.6E-91**	35.39	**1.4E-85**	1.38	1.9E-01
AT4G05020	NDB2	1.02	9.8E-01	21.68	**9.3E-11**	0.56	3.4E-01
AT2G41800	TEEBE (TEB)	5.45	**5.6E-04**	4.43	**1.2E-02**	2.03	2.4E-01
AT4G12490	AZI3	5.13	**5.7E-06**	4.34	**3.3E-04**	1.48	4.1E-01
AT1G65060	4CL3	4.05	**2.7E-08**	3.53	**5.9E-06**	1.16	6.9E-01
AT1G04600	XIA	3.00	**1.8E-03**	3.43	**2.0E-03**	2.77	**5.7E-03**
AT1G04220	KCS2	3.05	**1.4E-06**	3.40	**8.1E-07**	2.13	**2.7E-03**
AT5G08640	FLS1	3.59	**1.5E-08**	3.35	**9.7E-07**	1.01	9.9E-01
AT3G27400	PLL18	3.74	**5.5E-05**	3.15	**2.8E-03**	1.82	1.2E-01
AT1G60590	Pectin lyase-like superfamily protein	2.54	**3.9E-02**	2.99	**4.0E-02**	0.97	9.7E-01
AT4G12310	CYP706A5	3.28	**2.8E-14**	2.92	**8.0E-11**	1.30	1.8E-01
AT2G30766	FEP1	6.02	**1.2E-07**	2.61	**3.5E-02**	2.04	7.9E-02
AT5G58770	CPT4	3.85	**1.2E-08**	2.57	**8.9E-04**	1.76	**4.3E-02**
AT1G78290	SNRK2-8	3.83	**2.5E-13**	2.52	**1.6E-05**	1.26	3.7E-01
AT4G12480	EARLI1	3.04	**2.2E-06**	2.50	**8.9E-04**	1.34	3.4E-01
AT1G09240	NAS3	1.25	5.7E-01	2.49	**8.1E-03**	1.55	2.0E-01
AT2G36590	ProT3	1.98	**3.5E-02**	2.49	**1.3E-02**	1.83	7.6E-02
AT1G74010	Calcium-dependent phosphotriesterase superfamily protein	2.25	**9.2E-03**	2.40	**1.8E-02**	1.37	4.2E-01
AT5G37300	WSD1	3.12	**8.5E-05**	2.39	**1.7E-02**	1.66	1.4E-01
AT1G52342	hypothetical protein	3.64	**9.6E-09**	2.33	**2.5E-03**	1.00	9.9E-01

Including fold change values for each gene and each line for comparison. Statistical significance (FDR < 5%) indicated in bold. AOX1A-OEX: AOX1A overexpression line, Dual-OEX: AOX1A and NDB2 overexpression line, *ndb2*: NDB2 knockout line.

All of the other top 20 up-regulated transcripts in the dual-OEX line increased by only 2.3- to 4.4-fold and many of these were up-regulated to a larger degree in the AOX1A-OEX line (**Table 5**). These included several genes related to cell walls, lipids and membranes (often related to defense), secondary metabolite pathway genes and a stress-related *SUCROSE NON-FERMENTING 1-RELATED PROTEIN KINASE (SNRK2-8)* that is activated by salt, osmotic and drought stresses but also biotic stresses (Kim et al., 2012; Lee et al., 2015; Lei et al., 2020). The *FE-UPTAKE-INDUCING PEPTIDE1 (FEP1)* was also highly up-regulated in both dual-AOX and AOX1A-OEX lines. This protein is responsible for activating iron deficiency response genes, although no cognate downstream transcripts were significantly up-regulated.

Of the top-20 down-regulated transcripts in the dual-OEX line, only three were unique (**Table 6**). First, an unnamed gene typically expressed in the root cortex (Denyer et al., 2019). This gene is down-regulated in cold (Zhu et al., 2003) but up-regulated in cellulose synthase-deficient mutants as part of a defense response (Hernandez-Blanco et al., 2007). Second, a MtN21-like amino acid transporter family protein *USUALLY MULTIPLE ACIDS MOVE IN AND OUT TRANSPORTERS 33 (UMAMIT33)*. Third, a *QUA-QUINE STARCH (QQS)* gene, important for carbon-nitrogen balance (Li et al., 2015) and involved in pest defense (Qi et al., 2019).

**Table 6:** Top 20 down-regulated DEGs for dual AOX1A and NDB2 overexpression line relative to wild type.

Gene Locus	Gene Name/Description	AOX1A-OEX		Dual-OEX		*ndb2*	
		FC	FDR	FC	FDR	FC	FDR
AT3G47340	ASN1	0.13	**4.6E-47**	0.15	**4.2E-40**	0.66	**1.3E-02**
AT2G19800	MIOX2	0.10	**3.1E-04**	0.15	**1.3E-02**	0.33	1.3E-01
AT4G36850	PQ-loop repeat family protein/transmembrane family protein	0.16	**2.6E-26**	0.17	**5.6E-23**	0.51	**2.6E-04**
AT5G24770	VSP2	0.19	**1.4E-04**	0.19	**7.2E-04**	0.28	**6.1E-03**
AT5G20250	DIN10	0.18	**1.2E-07**	0.19	**1.9E-06**	0.48	**4.8E-02**
AT3G15450	aluminum induced protein with YGL and LRDR motifs	0.12	**4.2E-46**	0.19	**1.0E-27**	0.63	**7.2E-03**
AT1G62510	Expressed in the root cortex	0.53	1.9E-01	0.20	**1.5E-03**	0.82	7.4E-01
AT3G30775	ERD5	0.20	**1.8E-03**	0.20	**7.0E-03**	0.38	8.3E-02
AT3G09260	PYK10	0.31	**4.6E-03**	0.23	**1.3E-03**	0.37	**2.2E-02**
AT5G57550	XTH25	0.18	**3.1E-06**	0.25	**1.3E-03**	1.08	8.9E-01
AT2G20670	sugar phosphate exchanger, putative (DUF506)	0.21	**1.3E-41**	0.25	**2.1E-29**	0.89	4.3E-01
AT4G28040	UMAMIT33	0.56	1.3E-01	0.27	**2.7E-03**	0.87	7.8E-01
AT5G42800	DFR	0.27	**1.5E-03**	0.27	**6.5E-03**	0.17	**2.1E-05**
AT5G49360	BXL1	0.20	**1.8E-32**	0.28	**2.1E-19**	0.57	**2.2E-04**
AT4G35770	SEN1	0.23	**7.6E-12**	0.30	**1.3E-07**	0.92	8.1E-01
AT5G22920	RZPF34	0.36	**1.2E-09**	0.30	**1.4E-11**	0.61	**7.7E-03**
AT3G30720	QQS	0.53	9.5E-02	0.30	**4.9E-03**	1.05	9.3E-01
AT2G15880	LRX10	0.18	**5.2E-29**	0.30	**6.6E-14**	0.95	8.3E-01
AT5G41340	UBC4	0.33	**1.8E-16**	0.31	**7.4E-16**	1.10	6.0E-01
AT4G27450	HUP54	0.26	**3.6E-18**	0.33	**3.9E-11**	0.94	8.0E-01

Including fold change values for each gene and each line for comparison. Statistical significance (FDR < 5%) indicated in bold. AOX1A-OEX: AOX1A overexpression line, Dual-OEX: AOX1A and NDB2 overexpression line, *ndb*2: NDB2 knockout line.

Several of the other top 20 down-regulated transcripts in the dual-OEX line were related to energy metabolism: *GLUTAMATE-DEPENDENT ASPARAGINE SYNTHASE (ASN1)* and a glycosyl hydrolase family member *DARK INDUCIBLE 10 (DIN10)*, both of which may be suppressed by high sugar (Lam et al., 1994; Fujiki et al., 2001); a *PUTATIVE SUGAR PHOSPHATE EXCHANGER (DUF506)*; a *MYO-INOSITOL OXYGENASE (MIOX2)*; and *EARLY RESPONSIVE TO DEHYDRATION 5 (ERD5)*, which is a proline oxidase that is thought to be localised to the inner mitochondrial membrane, induced by oxidative stress and high L-proline concentrations and important for sustaining growth during stress (Verbruggen et al., 1996; Sharma et al., 2011). Other down-regulated transcripts were potentially involved in abiotic stress responses, including *VEGETATIVE STORAGE PROTEIN 2 (VSP2)*, *SENESCENCE 1 (SEN1)*; *RING ZINC-FINGER PROTEIN 34 (RZPF34)* and *HYPOXIA RESPONSE UNKNOWN PROTEIN 54 (HUP54)*. Also down-regulated in both AOX1A-OEX and dual-OEX lines were A beta-glucosidase, *XYLOGLUCAN ENDOTRANSGLUCOSYLASE/HYDROLASE 25 (XTH25)* and a bifunctional (beta)-D-xylosidase/(alpha)-L-arabinofuranosidase, all involved in loosening cell walls and secondary cell wall thickening.

### 3.4 Transcript levels of a select set of genes may be regulated by the AOX1A:NDB2 ratio

Genes were classified into a group called “regulated by AOX1A:NDB2”, if they fulfilled two conditions: their transcript must be either up- or down-regulated in the AOX1A-OEX line but expressed at wild type levels in the dual-OEX line; and the transcript must be commonly up- or down-regulated in both the AOX1A-OEX and *ndb2* lines. That is, DEGs that overlapped between the AOX1A-OEX and *ndb2* lines but not the dual-OEX line. This included 135 up-regulated transcripts (**Figure 2A**) and 74 down-regulated transcripts (**Figure 2B**), as listed in **Table S4**. Up-regulated transcripts could not be ascribed to particular biological processes using GO SLIM analysis, likely because a large proportion of these genes were either unnamed/hypothetical proteins or RNA molecules. On the other hand, down-regulated transcripts were strongly enriched for “photosynthesis” and “secondary metabolic process”.

Of the 209 genes in this group, 18 were chloroplast-encoded. Without exception, all chloroplast-encoded transcripts that were found to be down-regulated in AOX1A-OEX were also down-regulated in the *ndb2* line and unaffected in the dual-OEX line. This included several reaction center genes, ATP synthase subunits, a photosynthetic electron transfer transcript required for ATP synthesis, and the large subunit of RuBisCO (**Table S4**). This lends support to the idea that increasing AOX1A relative to NDB2 (either by over-expressing *AOX1A* or deleting expression of *NDB2*) drives a unique subset of transcriptional responses. In this case the down-regulated photosynthetic transcripts do not explain the growth phenotype of AOX1A-OEX, because the same changes were seen in *ndb2* in the absence of any growth phenotype. Previous studies with AOX1A knockdown or knockout lines highlighted the extramitochondrial effect of AOX1A manipulation on the expression of nuclear genes whose proteins localize to other cellular compartments (Umbach et al., 2005; Clifton et al., 2006; Giraud et al., 2008). Others have found that simultaneously inhibiting electron transport through mitochondrial complex IV and AOX leads to decreased transcription in the chloroplast (Zubo et al., 2014; Adamowicz-Skrzypkowska et al., 2020). Our results extend these observations, whereby enhanced AOX1A expression or loss of NDB2 expression in the mitochondria led to modified transcription of genes directly within the chloroplast.

Only 6 genes in this group were mitochondrially-encoded, including two Complex I subunits that were down-regulated in both AOX1A-OEX and *ndb2*. Together with the down-regulation of a nuclear-encoded anchor subunit of succinate dehydrogenase (*SDH3-2*), an imbalance in AOX1A:NDB2 may affect classical mitochondrial electron transport chain components *via* internal and retrograde signaling mechanisms. Two up-regulated calmodulin proteins, *CML47* and *CML23*, are also of interest because NDB2 activity is partially regulated by calcium and could be involved in calcium sensing or signaling (Geisler et al., 2007; Wagner et al., 2016). CML23 also regulates nitric oxide levels, which inhibits cytochrome c oxidase but not AOX (Millar and Day, 1996).

*AOX1A* and *NDB2* are commonly co-expressed in response to stresses (Clifton et al., 2005), therefore it is interesting that *RARE COLD-INDUCIBLE 2B (RCI2B)* and another protein of this family, *F-BOX STRESS INDUCED 3 (FBS3)*, as well as the cold-responsive *DREB1A*, are among the group of genes affected by the AOX1A:NDB2 ratio. Several transcription factors and hormone-responsive genes were also affected, many with roles in development and differentiation, including *ANGUSTOFOLIA 3 (AN3)*, *INFLORESCENCE DEFICIENT IN ABSCISSION-LIKE 2 (IDL2)*, *LITTLE ZIPPER 1 (ZPR1)*, *ARGOS* and *ARGOS-LIKE (ARL)*, and *TEOSINTE BRANCHED 1, CYCLOIDEA AND PCF (TCP3)*. A NEET group protein that is important for plant development, senescence, reactive oxygen homeostasis and Fe metabolism is interesting in relation to the delayed growth phenotype observed in the AOX1A-OEX line (Sweetman et al., 2019). The presence of *ABA4* in this set of genes was also relevant, as it is essential for neoxanthin biosynthesis and protection of photosystem II and there is building evidence that mitochondrial alternative respiration can protect photosynthetic activities during prolonged stress (Vanlerberghe et al., 2016). It should be noted also that expression of *AOX1A* in Arabidopsis is regulated by ABA response factors: in particular, *AOX1A* expression is repressed by the *ABSCISIC ACID INSENSITIVE 4 (ABI4)* transcription factor that also acts downstream of chloroplast retrograde signalling pathways (Giraud et al., 2009). The large number of photosynthesis-related genes belonging to this group suggests that altering the AOX1A:NDB2 ratio has a dramatic effect on the composition of the photosynthetic apparatus and presumably photosynthetis in the leaves.

### 3.5 Functional responses to AOX1A and NDB2 manipulation

Increased transcript levels of *AOX1A* and *NDB2* confirmed that these genes were indeed overexpressed in the AOX1A-OEX and dual-OEX lines, by at least 35-fold and 20-fold, respectively (**Table S5**). However, there were no significant changes in other alternative pathway transcripts in any line, relative to wild type.

#### 3.5.1 Overviews of metabolism, cellular responses and regulation

To gauge the overall effect of altered *AOX1A* and *NDB2* expression on various processes and metabolic pathways, the data were used to populate “overview” MapMan maps (Thimm et al., 2004). As mentioned previously, many transcriptional changes were observed in the AOX1A-OEX line, and these either returned to normal in the dual-OEX line, or were retained (but often only partially), while the *ndb2* line also shared many transcriptional effects with the AOX1A-OEX line. These responses are clearly demonstrated in “Metabolism Overview” maps (**Figure S2**), especially under the sub-sections of “Light Reactions”, “Tetrapyrrole”, “minor CHO” and “Cell wall”.

The “Regulation Overview” map (**Figure S3**) shows that many transcription factors were up- and down-regulated in the AOX1A-OEX line, but far fewer were altered in the dual-OEX line. Of the hormone pathways, the AOX1A-OEX line showed general up-regulation of genes related to SA and ABA, and general down-regulation of genes related to GA, cytokinins and JA. IAA and Ethylene-related genes were both up- and down-regulated in AOX1A-OEX. The down-regulated IAA-related genes were conserved in dual-OEX, suggesting that these genes may respond to increased AOX1A regardless of NDB2 expression. The story was quite different for the *ndb2* line, which showed a clear up-regulation of genes related to protein degradation, receptor kinases and calcium regulation. This may suggest a greater sensitivity to external stimuli such as pathogens, consistent with the “Cellular Response Overview” map (**Figure S4**), which showed remarkable up-regulation of genes involved in biotic stress and heat stress responses. There were also strong up- and down-regulation of genes involved in biotic stress, plant development, heat and drought/salt stress in the AOX1A-OEX line (**Figure S4**). Similar profiles were seen in the dual-OEX line, but again, these were generally fewer and less pronounced. The *ndb2* line again shared similarities with the AOX1A-OEX line, particularly for drought/salt stress and “Misc. abiotic stress”.

#### 3.5.2 Mitochondrial electron transport chain components

A decrease in ATP synthesis might be expected in the AOX1A-OEX line, not only due to a smaller proton motive force across the inner mitochondrial membrane as a result of enhanced AOX1A (assuming it is active), but also *via* up-regulation of mitochondrial *ATP SYNTHASE INHIBITOR FACTOR 1 (IF1)* and *via* down-regulation of Complex I and II subunit transcripts (*NAD9*, *NAD4*, *SDH3-2*) (**Table S6**; **Figure S5**). The *ndb2* line showed a decrease in the same Complex I and II transcripts, but no effect on ATP synthase nor its inhibitor was evident. Intriguingly, there was also a 2.6-fold induction of a putative mitochondrial phosphate transporter in the *ndb2* line. Further studies with purified mitochondria from these lines together with oxygen discrimination and flux analyses are necessary to determine whether there are any changes to mitochondrial activities *in vivo*.

#### 3.5.3 Primary respiratory pathways and carbon usage

Transcripts of sugar- suppressed genes *ASN1*, *DIN10* and *MYO-INOSITOL OXYGENASE 2 (MIOX2)* were strongly down-regulated in both the AOX1A-OEX and dual-OEX lines and featured in the top 20 down-regulated DEGs of the dual-OEX line. Other transcripts related to sugar metabolism, transport and regulation (*SWEET1*, *2*, *10* and *AKINBETA1*) were also affected although some of these occurred only in the AOX1A-OEX line. These changes may reflect an increase in mobilisation of stored carbohydrates, perhaps in response to decreased ATP synthesis in the mitochondria (see above). However, these genes cannot explain the different growth responses because most changes were observed in both the AOX1A-OEX and dual-OEX lines.

Transcripts of core glycolytic and TCA cycle enzymes were generally unaffected (**Figures S6, S7**, **Table S7**), consistent with previous studies with *AOX1A* antisense plants (Umbach et al., 2005; Clifton et al., 2006). This may indicate that glycolysis and the TCA cycle do not rate limit sugar metabolism under our growth conditions. However, strong and opposing regulation of phosphoglycerate mutase isoforms were seen, with one putative isoform down-regulated in the AOX1A-OEX line (by 99%) and another isoform up-regulated in the *ndb2* line (7-fold). Phosphoglycerate mutase catalyses a reversible, non-rate-limiting step but transcript level changes of this magnitude could affect carbohydrate metabolism.

#### 3.5.4 Photosynthesis

Several photosynthesis-related ontologies were significantly affected in the AOX1A-OEX line, including members of light harvesting complexes for photosystem I and II, chlorophyll-binding proteins, reaction centres, antennae systems, electron transfer mediators, complex assembly proteins and RuBisCO subunits (**Table S8**). The MapMan “Photosynthesis” map (**Figure S8**) also indicated strong down-regulation of eleven PSI and PSII light-harvesting complex genes in the AOX1A-OEX line as well as two PSI reaction center genes. Five PSII reaction center genes were down-regulated, although another four were up-regulated. The J subunit of PSI was also up-regulated, as well as a protein required for assembly of the thylakoid NAD(P)H complex, *PQL3*. At least one PSII light-harvesting gene and several PSI and PSII reaction centre genes were among the few transcripts that showed a conserved response in the AOX1A-OEX and dual-OEX lines but not in the *ndb2* line. Therefore, while many photosynthesis-related genes may be responsive to changes in AOX1A:NDB2, the impact of overexpression of AOX1A alone on some photosynthetic genes was not reversed by rebalancing AOX1A and NDB2 expression (**Figure S8**). If these transcript changes cause changes in protein abundance, we might have expected plant growth to be more affected than previously observed in these lines (see Sweetman et al., 2019); perhaps photosynthesis was not limiting under our growth conditions. On the other hand, decreased expression of light-harvesting complexes is consistent with the ability of the AOX1A-OEX and the dual over expressing lines to better tolerate an increase in light intensity (Sweetman et al., 2019).

Knockout *aox1a* lines have impaired PSII function, especially under high light and drought conditions (Bartoli et al., 2006; Giraud et al., 2008; Yoshida et al., 2011). Watanabe et al. (2016) suggested that when the cytochrome pathway becomes limited or inhibited, excess reductants can flow through the AP, minimising excess ROS/RNS production. Zhang et al. (2011) suggested that AOX inhibition leads to build-up of excess reducing equivalents and PSI becomes over-reduced. Our experiments suggest that overexpression of AOX1A can alter signalling that affects the expression of both PSI and PSII in the nucleus and chloroplast, and that this effect can be largely (but not completely) reversed if NDB2 is overexpressed as well (**Figure S8**). Overexpression of both NDB2 and AOX1A together could allow more rapid oxidation of excess reducing power exported from the chloroplast, especially under photo-inhibitory conditions such as combined drought and high light stress.

#### 3.5.5 Antioxidants and osmoprotectants

In a previous study, AOX-overexpressing lines of *A. thaliana* (S5 and S9) showed higher ascorbate content in leaf extracts, and enhanced production of ascorbate in detached leaves and isolated mitochondria (Bartoli et al., 2006). Galactonolactone dehydrogenase (GLDH) is the only mitochondrial enzyme of the ascorbate biosynthesis pathway, while GDP-L-galactose phosphorylase and the others are dual localized to the cytosol and nucleus (Fenech et al., 2021). Furthermore, GLDH associates with Complex I of the mETC, and donates electrons directly to cytochrome c during the catalysis of galactonolactone to ascorbate (Millar et al., 2003). While the transcript and activity of GLDH was unchanged in AOX1A-overexpressing lines (**Table S9**; Bartoli et al., 2006), an increase in AOX activity might increase the availability of oxidized cytochrome c. Interestingly, in the present study, the gene encoding GDP-L-galactose phosphorylase (*VITAMIN C DEFECTIVE 2; VTC2*), was strongly up-regulated in AOX1A-OEX. This is a potentially rate-limiting step (Dowdle et al., 2007; Fenech et al., 2021) and enhanced *VTC2* transcript in AOX1A-OEX could lead to increased ascorbate biosynthesis. Given the increase in ascorbate levels and *VTC2* transcript in separate AOX1A-OEX lines, it is possible that over-expression of AOX1A can drive increased ascorbate production. Meanwhile in the *ndb2* line, an ascorbate oxidase gene involved in the oxidation of ascorbate to monodehydroascorbate was strongly up-regulated, suggesting an increase in oxidative stress signaling.

Strong up-regulation of transcripts encoding two proline transporters (*PROT2* and *3*) and a membrane protein potentially involved in drought-induced proline accumulation (*AFL1*; (Kumar et al., 2015), might suggest accumulation of proline in the AOX1A-OEX and dual-OEX lines (**Table S9**). This is supported by decreased transcript levels of proline catabolism enzymes, the mitochondrial *PROLINE DEHYDROGENASE 1* (*PRODH1*), also known as *ERD5* (Kiyosue et al., 1996) and *PRODH2*, which is colocalized to the mitochondria and chloroplasts (Van Aken et al., 2009). However, the *ERD5* promoter contains a proline-inducible element (Nakashima et al., 1998), therefore down-regulation of this transcript could also point to decreased availability of proline in the cell. Recently, it was revealed that AOX activity can facilitate the catabolism of stress-accumulated proline (Oh et al., 2021), and therefore a lower level of available proline might be expected in plants over-expressing AOX1A.

#### 3.5.6 Secondary metabolism – flavonoids, pigments, phenylpropanoid pathway

**Figure 4:**
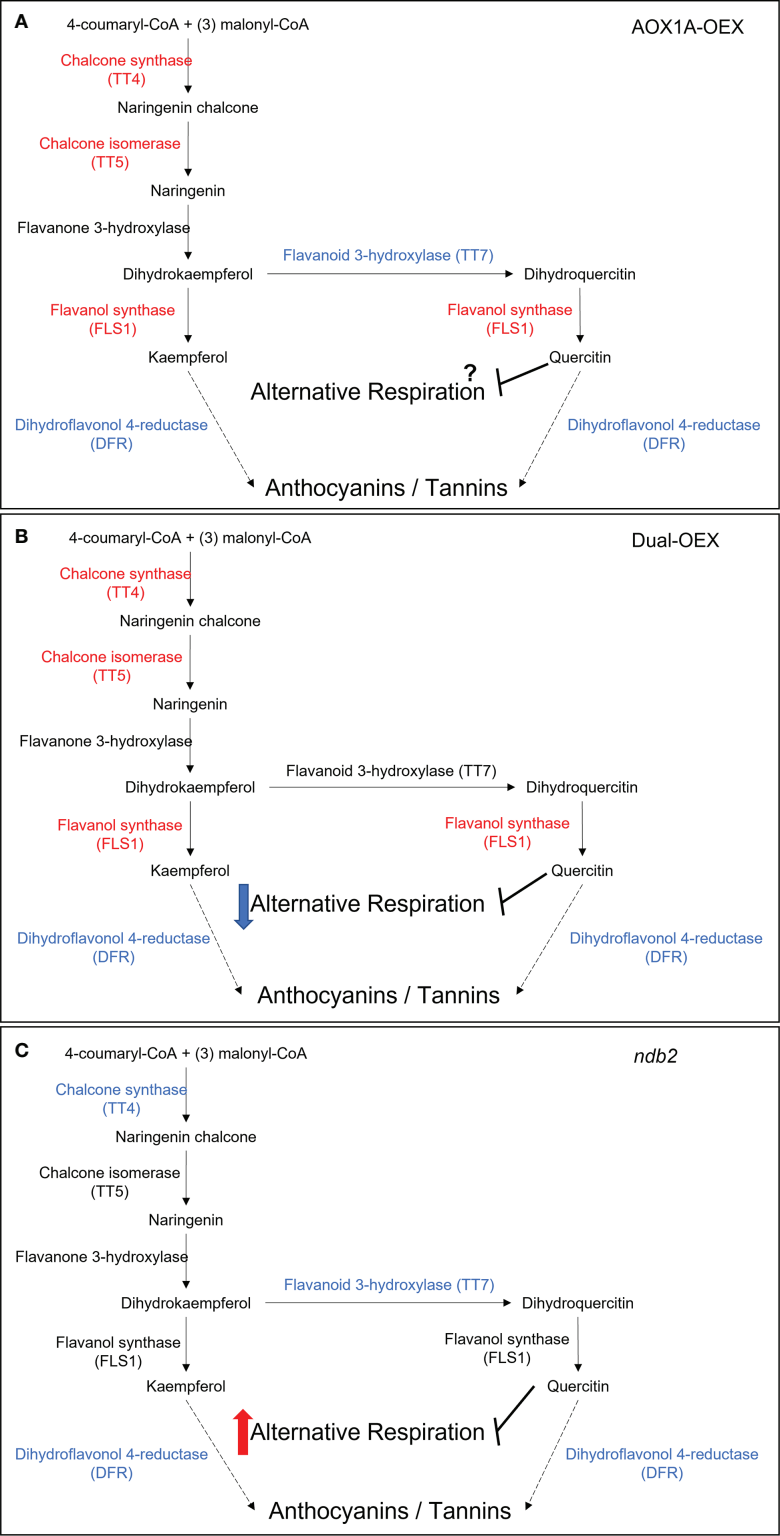
Flavonoid biosynthesis pathways and the potential effects on alternative pathway regulation in **(A)** AOX1A overexpression line, **(B)** dual overexpression line and **(C)** NDB2 knockout line. Major metabolite and enzyme steps in black. Red indicates an up-regulated transcript, blue indicates a down-regulated transcript. Red and Blue arrows represent potential down- and up-regulation of AOX activity. Dotted arrow indicates a multiple-step pathway with details omitted.

Both the AOX1A-OEX and dual-OEX lines showed increased expression of genes related to chalcones and flavonols, with a down-regulation of anthocyanin metabolism genes (**Figure S9**). The *ndb2* line showed fewer changes but strong up- and down-regulation of dihydroflavonol- and anthocyanin-related transcripts. At the gene ontology level, flavonoid biosynthetic process was over-represented in all three lines compared to wild type. Representative genes of this group included *CHIL*, *FLS1*, *TT4*, *TT5*, *UGT84A2*, *FLA15* and *PDE339*. *FLS1* (*FLAVONE SYNTHASE 1*) was one of the most significantly altered transcripts in the dual OEX line. Balance between *TT4* (a chalcone synthase) and *TT7* (a flavonoid 3-hydroxylase) can determine the relative abundance of quercitin and kaempferol in Arabidopsis (Lewis et al., 2011). This may be relevant considering the inhibitory effects of quercitin, but not kaempferol, on alternative respiration (Shimoji and Yamasaki, 2005). The increase in *TT4* transcript in both AOX1A-OEX and dual-OEX lines (and in a separate AOX1A overexpression line; Fiorani et al., 2005) may increase kaempferol biosynthesis as a result of elevated AOX. Meanwhile, a decrease in *TT7* transcript only in the AOX1A-OEX line, suggests that the inhibitory role of quercitin, if indeed it occurs *in vivo*, may be by-passed when AOX1A is overexpressed, but potentially regained when NDB2 and AOX1A levels are rebalanced (**Figure 4**). This raises the possibility that *in vivo* AOX1A activity may be greater in the AOX1A-OEX line relative to the dual overexpression line, and this in turn may directly affect energy state and growth in the AOX1A-OEX line. This requires further experimentation using oxygen discrimination to determine AOX activity. Transcripts of *DIHYDROFLAVONOL 4-REDUCTASE (DFR)* and *LEUCOANTHOCYANIDIN DIOXYGENASE (LDOX)* were down-regulated strongly in all three lines, suggesting decreased capacity for further metabolism of kaempferol (e.g. to anthocyanins). Based on these transcriptional changes, levels of kaempferol may be higher in both lines overexpressing AOX1A, which could ready the cell for rapid biosynthesis of quercitin and inhibition of the alternative respiration, as necessary.

Flavonoids often accumulate in response to abiotic or biotic stresses, to assist the scavenging of ROS that escape from the chloroplast or mitochondria during oxidative stress, thereby improving tolerance to stress (Fini et al., 2011; Nakabayashi et al., 2014). This may explain the very strong upregulation of flavonoid and anthocyanin-related transcripts in *aox1a* lines exposed to a combined drought and light stress (Giraud et al., 2008), which coincided with increased anthocyanin content, but does not explain the accumulation of transcripts when AOX1A is overexpressed in the absence of a stress (**Figure S9**). It is feasible that the regulators of flavonoid genes, including MYB transcription factors, which are known to be affected by redox status (Dubos et al., 2010), including photosynthetic redox status (Akhtar et al., 2010), may also be responsive to changes in the expression of alternative respiration pathway components. Transcript levels of several MYB transcription factors, including *MYB111*, which is known to positively affect flavonol biosynthesis (Stracke et al., 2007) were significantly up-regulated in AOX1A-OEX and dual-OEX, giving credence to this hypothesis.

Phenylpropanoid biosynthesis genes were up- and down-regulated in the AOX1A-OEX line and also, but to a lesser extent, in the dual-OEX line (**Figure S10**). Strongly up-regulated transcripts included a feruloyl-CoA transferase (*RWP1*), two cinnamyl alcohol dehydrogenases (*CAD4* and *CAD9*), a fatty acid reductase (*FAR5*), a 3-ketoacyl-CoA synthase (*KCS2*) and a 4-coumarate:CoA ligase (*4CL3*). The latter was also one of the most strongly up-regulated transcripts in the dual-OEX line. Sharma et al. (2019) suggested that the role of *4CL3* was to alter the activity and transcription of numerous genes in the phenylpropanoid pathway. Links between AOX and phenylpropanoid metabolism have been demonstrated previously (Macedo et al., 2012; Sircar et al., 2012). Many of the genes involved in the phenylpropanoid pathway are stress responsive and react to a broad range of stressors (Dixon and Paiva, 1995; Sharma et al., 2019). Alteration to the alternative pathway of respiration, with its broad ranging effects, may modify the signaling between mitochondria and chloroplasts, resulting in adjustments to downstream secondary metabolite pathways such as flavonoid and phenylpropanoid biosynthesis. This also may explain, in part, why these plants were able to recover when exposed to a combined moderate light and drought stress (Sweetman et al., 2019).

#### 3.5.7 Hormone metabolism/regulation

Transcripts of genes involved in various hormone biosynthesis and regulation pathways were affected, particularly in the AOX1A-OEX and *ndb2* lines (**Figure S3**). However, the transcriptional similarities typically observed between AOX1A-OEX and *ndb2* in other pathways, were less conserved here. For example, the AOX1A-OEX line showed strong down-regulation of abscisic acid (ABA) biosynthesis genes and up-regulation of the ABA catabolism gene, *CYP707A1* (**Table S10**), supporting the negative relationship between AOX1A and ABA, while *ndb2* showed little regulation of the same transcripts. For the jasmonate biosynthesis pathway, AOX1A-OEX showed strong down-regulation of several transcripts, while *ndb2* did not (**Table S10**, **Figure S3**). This suggests that the overexpression of AOX1A and knockdown of NDB2 may lead to different hormonal signals despite similar transcriptional responses in other pathways.

Transcripts of several genes involved in ethylene metabolism and signaling, salicylic acid metabolism and responses, auxin transport and responses, gibberellin biosynthesis and modifications, ABA biosynthesis and jasmonate biosynthesis, were variously up- and down-regulated (**Figure S3**; **Table S10**). Overall, increased AOX1A expression may (i) influence ethylene- and jasmonate-directed ROS responses, cell death and root morphology pathways (ii) induce the accumulation of SA *via* ROS signaling (Zhang et al., 2012), which may sacrifice growth to up-regulate stress priming mechanisms, (iii) support the antagonistic relationship previously observed between auxin signaling and mitochondrial signaling for *AOX1A* expression (Ivanova et al., 2014), and (iv) lead to a general increase in gibberellins but a proportional decrease in GA4, which is important for shoot elongation and flower initiation (Eriksson et al., 2006). Many of these factors could contribute to the delayed growth phenotype of AOX1A-OEX (Sweetman et al., 2019), but are yet to be tested experimentally.

#### 3.5.8 Stress responses

A defensin-like protein showed the strongest up-regulation of any transcript in the AOX1A-OEX line, and numerous other transcripts related to abiotic and biotic stress featured in the top 20 up- and down-regulated transcript lists (**Figure S11**; **Tables 1**, **2**). Some examples are given below, and in many cases these changes were absent, or at least dampened in the dual-OEX line. Most notable, though, was the response of the *ndb2* line, with a striking up-regulation of transcripts relating to abiotic and biotic stress, R proteins, PR proteins, heat shock and signaling (**Figures S4**, **S11**) and receptor kinases (**Figures S3**, **S12**). These receptor kinases, particularly DUF26 members, have been implicated in biotic and abiotic stress responses and are regulated by growth effectors such as SA (Wrzaczek et al., 2010). Considering the vulnerability of *ndb2* plants to a combined drought and moderate light treatment (Sweetman et al., 2019), this up-regulation of stress response pathways under standard growth conditions does not appear to improve tolerance to stress.

**Figure 5:**
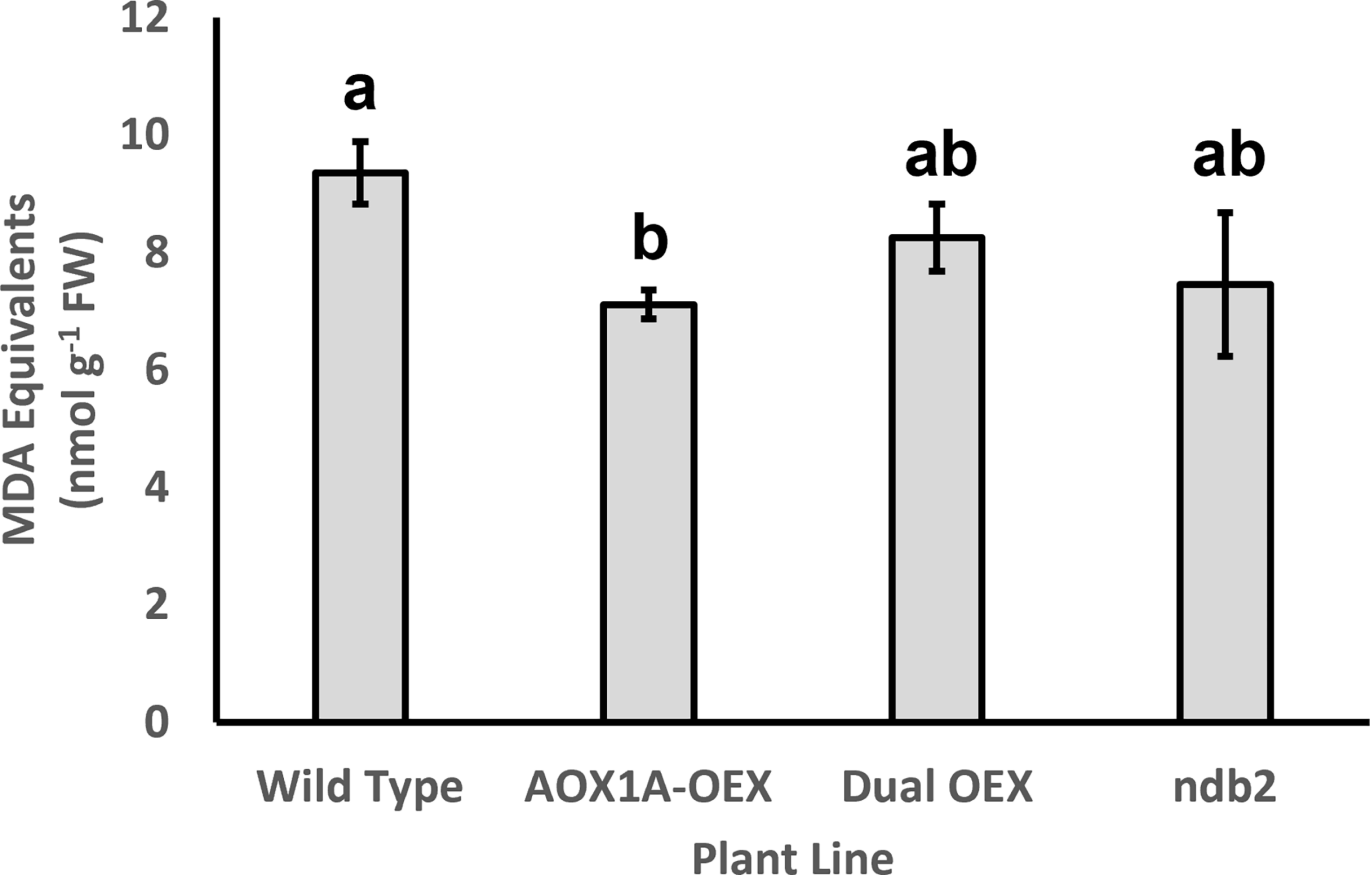
Effect of altered AOX1A and NDB2 expression on lipid peroxidation levels. Malondialdehyde equivalents were measured using a TBARS assay (Hodges et al., 1999), from aliquots of the powdered leaf samples used for RNA extractions. Columns represent means (n = 3 ± S.D.). Statistical significance indicated by different letters, based on one-way ANOVA with *post-hoc* Bonferonni-corrected pairwise comparison (corrected p < 0.05).

A major role proposed for AOX is in the prevention of ROS production during exposure to environmental stress (Maxwell et al., 1999; Vanlerberghe, 2013). Therefore, it was not surprising to see strong down-regulation of several peroxidase genes in the AOX1A-OEX line, including a 97% decrease in *PEROXIDASE9 (PRX9)* transcript. Transcription factors such as *REDOX RESPONSIVE TRANSCRIPTION FACTOR 1 (RRTF1)*, *ETHYLENE-RESPONSIVE ELEMENT BINDING PROTEIN (EBP)* and *ETHYLENE RESPONSIVE ELEMENT BINDING FACTOR 6 (ERF6)* respond to ROS and mitigate oxidative stress by decreasing ROS levels, ultimately also protecting against cell death. Each of these transcripts was strongly down-regulated in the AOX1A-OEX line. That is, AOX1A overexpression led to altered ROS-signaling networks, likely *via* a decrease in ROS generation in the mitochondria. Indeed, there was a significant decrease in levels of malondialdehyde equivalents in the AOX1A-OEX line, suggesting a decrease in ROS-related lipid peroxidation (**Figure 5**). The dual-OEX and *ndb2* lines also showed similar decreases but were not statistically significant (**Figure 5**).

Several stress-related transcripts that were up-regulated in the AOX1A-OEX line also have roles in the regulation of growth and morphology. For example (1) a Cytochrome P450 involved directly in ABA catabolism and post-germination growth (*CYP707A1*; Okamoto et al., 2006), (2) a TF involved in integrating light and hormone signaling to regulate internode elongation (*ATH1*; Ejaz et al., 2021), (3) a coiled-coil protein involved in chloroplast movement in response to excess light (*PMI2*; Kodama et al., 2010), (4) a tandem zinc knuckle protein that negatively regulates hypocotyl growth in the mornings (*TZP*; (Loudet et al., 2008), and (5) all four “suppressor of phyA-105” protein family genes (*SPA1-4*), which suppress photomorphogenesis in seedlings and elongation in mature plants (Laubinger et al., 2004). These changes could contribute to both the stress tolerance feature of AOX1A-OEX lines and the delayed growth phenotype observed under conventional growth conditions (Sweetman et al., 2019).

#### 3.5.9 Biotic stress

Glucosinolates in the Brassicales order are typically associated with biotic stress defence and innate immune response (Sonderby et al., 2010). Glucosinolate biosynthesis genes were significantly downregulated and “glucosinolate metabolic process” featured in GO profiling for down-regulated transcripts in the AOX1A-OEX and *ndb2* lines (**Table S12**). An antagonistic relationship between AOX and glucosinolates has been hypothesised before, whereby repression of AOX by glucosinolates could either enable mitochondrial ROS accumulation, triggering a defense response *via* mitochondrial retrograde signaling pathways, or otherwise enable prioritisation of alternative stress responses (Zhang et al., 2017). Similarities between the transcriptome responses to mitochondrial perturbation and biotic stress have also been noted previously (Clifton et al., 2006; Schwarzlander et al., 2012; Umbach et al., 2012).

Altered cell wall biochemistry can be another indicator of altered biotic stress responses. Several transcripts relating to cell walls were up-regulated in the AOX1A-OEX line, and in many cases the *ndb2* line, including a polygalacturonase protein required for release of cell-wall-derived PR elicitors (*ADPG1*; Gallego-Giraldo et al., 2020), a pectate lyase involved in the response to nematodes (*PLL18*; Wieczorek et al., 2014) and a DUF642 cell wall protein involved in cell replication and hypocotyl cell elongation (*TEB*; Salazar-Iribe et al., 2016). These responses, as well as the strong down-regulation of a gene involved in converting myo-inositol to D-glucuronic acid (*MIOX2*), suggests that there may be an increase in cell wall degradation and a decrease in the synthesis of new cell wall material in these lines.

#### 3.5.10 AOX1A regulators and signaling pathways

**Figure 6:**
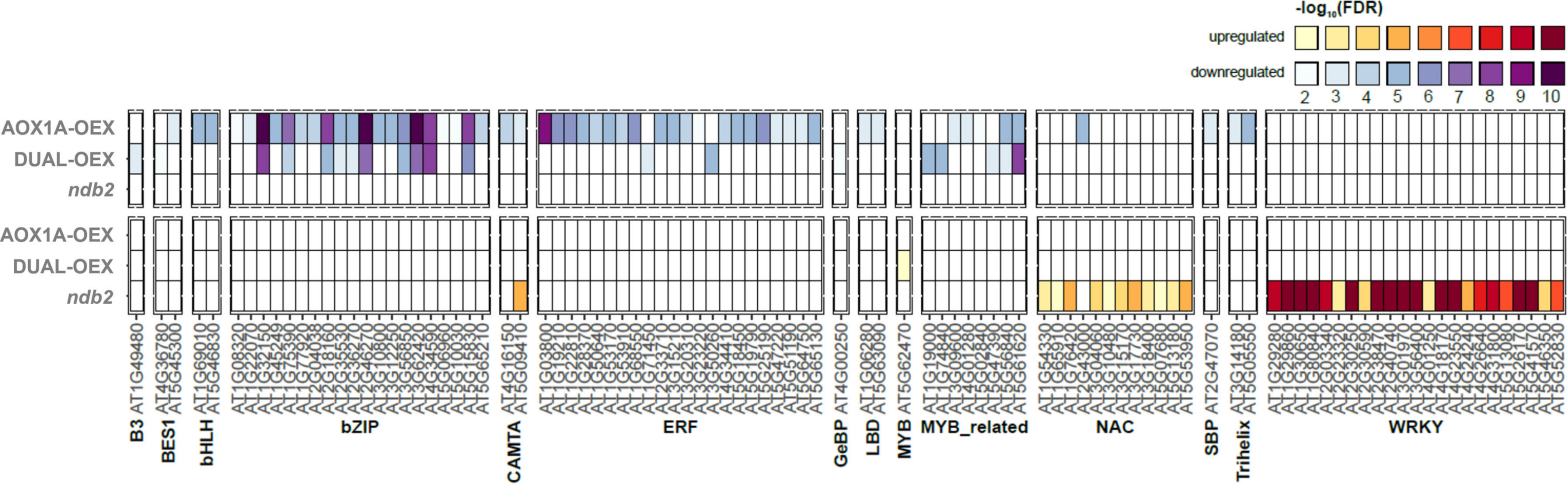
Enrichment of transcription factors based on differential expression of target genes. EAT-UpTF was used to profile the enrichment of transcription factor target genes among the DEGs of each line, relative to wild type. greater colour intensity represents greater significance (-log_10_FDR). Transcription factors were enriched in both down-regulated (top) and up-regulated (bottom) DEGs.

WRKY transcription factors have been suggested as a nexus for mitochondrial and chloroplast stress responses (Van Aken and Whelan, 2012). WRKY40 in particular, has been recognized as a negative regulator of *AtAOX1A* and more generally as a modulator of stress-responsive genes that are encoded in the nucleus but whose proteins localize to the mitochondria or chloroplast (Van Aken et al., 2013). Transcript levels of *WRKY40* and one of its targets, *REDOX-REGULATED TRANSCRIPTION FACTOR (RRTF1)*, were both strongly down-regulated in the AOX1A-OEX line. This implies a decrease in stress-related ROS accumulation in the AOX1A-OEX line and a release of *AOX1A* repression. *RRTF1* was one of five ethylene responsive factors that were strongly down-regulated in AOX1A-OEX (**Table S10**), which also included *ERF4* and *ERF11*. Target genes of these transcription factors were enriched among down-regulated transcripts of the AOX1A-OEX line (**Figure 6**), therefore the decrease in WRKY40, RRTF1, ERF4 and ERF11 transcripts likely resulted in a decrease in their protein level and activity.

The *ndb2* line also showed enhanced levels of transcripts relating to transcriptional control (**Figure S13**), particularly WRKY TFs. *WRKY40*, it’s binding partner *WRKY18* and the target genes of these two (and other WRKY targets) were strongly up-regulated in the *ndb2* line (**Figure 6**, **Table S13**), suggesting that NDB2 also can play a role in mitochondrial retrograde signaling. The *WRKY40* gene is reportedly induced by pathogens including *Pseudomonas syringae* and *Botrytis cinerea* (Xu et al., 2006; Abeysinghe et al., 2019), and this transcript represents a rare example of opposing transcriptional responses between the AOX1A-OEX and *ndb2* lines. While repression of AOX1A transcription by WRKY40 in the *ndb2* line could be interpreted as an attempt to recreate balance between *AOX1A* and *NDB2*, there was no significant change in *AOX1A* transcript level in this line.

Many of the stress-responsive mitochondrial genes contain multiple WRKY binding sites within their promoters (Van Aken et al., 2013). In the *ndb2* line, there was a 12-fold increase in *WRKY30* (among the top 20 DEGs for the *ndb2* line) and 2-3-fold increases in *WRKY46*, *47*, *48* and *53*. Target genes of many WRKY TFs were also strongly enriched in the up-regulated DEGs of the *ndb2* line (**Figure 6**) indicating that the WRKY transcription factors were functional. Many WRKYs are responsive to oxidative stress, including *WWRKY30*, *46* and *48* (Scarpeci et al., 2008) and some can provide oxidative stress and salinity tolerance when overexpressed (Scarpeci et al., 2013). However, there was no evidence of oxidative stress in the *ndb2* line under our standard growth conditions (**Figure 5**).

Transcript levels of AOX regulators *NAC017* and *NAC013* (De Clercq et al., 2013; Ng et al., 2013), their negative regulator *RCD1* (Shapiguzov et al., 2019; Wang et al., 2020) and positive regulators *CDKE;1* and *KIN10* (Ng et al., 2013) remained unchanged in all lines. However, many other transcriptional regulators were affected in the AOX1A-OEX line, including down-regulation of EREBP, GRAS, HB, C2C2-CO-like, C3H and MADS transcription factors. *CEJ1*, which is an ethylene- and jasmonic acid-responsive DREB involved in defense and freezing stress responses, also showed down-regulation in transcript levels of both itself and its target genes. These ethylene response factors may be important for regulating the transcriptional responses to AOX1A overexpression. Any of these transcription factors and their target genes could also be involved in the delayed growth phenotype of the AOX1A-OEX line.

Several bZIP transcription factors were enriched in the down-regulated DEGs of the AOX1a-OEX line (**Figure 6**). This included almost the entire S1 subgroup of bZIPs (i.e. *bZIP2*, *11*, *44* and *53*), which can regulate key metabolic genes in response to cellular energy status, by forming heterodimers with group C bZIPs (for a recent review, see Droge-Laser and Weiste, 2018). Transcript levels of group C genes including BZIP*63* were directly down-regulated, as well as several downstream targets of bZIP63 including e.g. *DREB1A*, *ASN1*, *ERD5*, *DIN10* (**Table S11**; Baena-Gonzalez et al., 2007; Dietrich et al., 2011; Matiolli et al., 2011). bZIP63 is activated by phosphorylation through the activity of the *SUCROSE NON-FERMENTING 1-RELATED KINASE 1 (SnRK1)*, whose regulatory subunit *AKINbeta1* was also down-regulated in the AOX1A-OEX line. Overall, this suggests that the plant signaling network is responding to a high-energy status within the cell (i.e. high levels of free sugars) but this is unlikely to drive the delayed growth phenotype as discussed in a previous section. It is also possible that some of these genes respond to factors other than sugars or energy status, such as ROS-induced signals.

Overall, our results suggest that altered AOX1A and NDB2 expression causes changes in signaling pathways that extend far beyond the mitochondrion, resulting in many transcriptional changes. While some of these changes were shared between lines, many were also unique to each line, highlighting complexities in retrograde signaling that require further investigation.

#### 3.5.11 Comparison of lines with differentially expressed AOX1A and NDB2

To date, we have compared transcriptome changes in lines overexpressing either AOX1A alone or with NDB2, as well as an *ndb2* line, under standard growth conditions. The se lines behave quite differently when subjected to environmental stresses such as high light and drought (Sweetman et al., 2019): the AOX1A-OEX and dual-OEX lines show improved resilience, while the *ndb2* line is more sensitive. Previous studies have focused on the transcriptomic responses of *aox1a* plants (e.g. Umbach et al., 2005; Giraud et al., 2008), rather than AOX1A-overexpressing plants. One manuscript has assessed the transcriptome profile of rapeseed seedlings over-expressing a typically seed-specific AOX1B gene, where it was found that transcript levels of stress-related genes were both up- and down- regulated, including genes involved in ABA, oxidative stress and osmotic stress responses, and the over-expressing plants were hypersensitive to ABA (Yang et al., 2019). Here in Arabidopsis, alterations in AOX1a and NDB2 expression also led to transcriptional changes of genes involved in stress responses and ABA. Over-expression of AOX1A led to downregulation of transcripts involved in ABA and JA biosynthesis, ABA-regulated bZIP transcription factors and ethylene response factors. Meanwhile, knockout of NDB2 led to up-regulation of WRKY and NAC signalling pathways that may involve an increase in abscisic acid and ethylene. Decreased functionality of ethylene response factors and bZIP TFs in the AOX1A-OEX line, and to a lesser degree in the dual-OEX line, suggests a general decrease in ethylene and abscisic acid signaling pathways in response to AOX1A overexpression that is somewhat reversed upon rebalancing AOX1A and NDB2 levels. Exposing plants of the AOX1A-OEX line to ethylene or ABA might therefore prevent some of the transcriptional changes, and potentially reverse the growth phenotype effect. Clearly, further growth analyses under various environmental conditions are needed.

WRKY40 was one of the few transcripts that showed opposing responses between AOX1A-OEX and *ndb2* lines, and could be a significant point of difference, considering the importance of this transcription factor in stress signaling pathways (Van Aken et al., 2013). Knockout of NDB2 also led to a notable up-regulation of transcripts involved in biotic stress responses.

Our results suggest that changing the ratio of AOX expression to that of NDB2 has a particularly pronounced transcriptional response, and that the two enzyme activities need to be balanced to preserve wild-type gene expression patterns. The AOX1A-OEX line had over four times the number of significantly altered genes (relative to wild type), compared to the dual AOX1A and NDB2 overexpression line. The most likely explanation is that changes caused by overexpression of *AOX1a* alone were attenuated by co-overexpression of *NDB2.* This agrees with phenotyping data where growth delays seen in the single OEX were no longer present in the dual overexpression lines (Sweetman et al., 2019) and may also be related to altered ROS metabolism, based on MDA equivalents (**Figure 5**). Meanwhile, a subset of transcripts behaved similarly in both the AOX1A-OEX line and the *ndb2* line, suggesting some common adjustment when AOX1A expression is high relative to NDB2.

Rebalancing the input (i.e. NDB2) and output (AOX1A) of the electron transport chain seems important for maintaining regular growth during non-limiting conditions while maintaining the potential stress benefit of an enhanced alternative respiration pathway. Altering this balance is likely to affect not only mitochondrial redox poise and ROS accumulation, but also the redox poise of other cell compartments. For example, decreasing the capacity to oxidise NADH generated by photorespiration of glycine, or excess reducing equivalents exported from the chloroplasts, could lead to over reduction of the chloroplast and accumulation of reactive oxygen species in the cell. This can certainly explain the sensitivity of the *ndb2* and *aox1a* lines to photoinhibitory stress conditions (Giraud et al., 2008; Watanabe et al., 2016; Sweetman et al., 2019; Li et al., 2020). Redox regulation has been demonstrated for several transcription factors, affecting DNA binding and cellular distribution (Tron et al., 2002; Heine et al., 2004; Dietz, 2014). As an example, group G bZIPs such as bZIP16 contain a conserved cysteine residue which, when reduced, enables binding and transcriptional activation of the target DNA (Shaikhali et al., 2012). In AOX1A overexpressing plants, target genes from three such transcription factors, including bZIP16, were significantly down regulated despite little or no change in expression of the transcription factors themselves. As such, decreased transcript levels of the PSII light harvesting complex gene *LHCB2.4* and other target genes in the AOX overexpression lines might be due to decreased binding efficiency of bZIP16 as a result of altered redox poise of the cell. Further comparisons between control and stressed plants may help to elucidate the role of bZIP16, WRKY40 and other signaling molecules in the altered stress tolerance of these lines.

It should be noted that some transcriptional changes observed between lines may be due to their proximity to transgene insertion sites, which are unknown for the AOX1A-OEX background and dual-OEX lines. However, at least three individual dual-OEX lines showed the same growth phenotype (Sweetman et al., 2019), therefore, if there is an insertional effect in dual-OEX, it is unlikely to affect growth. We can also assume that any transcriptional change in AOX1A-OEX that reverted towards WT expression in the dual-OEX line, is not due to an insertional effect in either line. Nevertheless, targeted experimental analyses are required to define the origin and downstream effects of the key transcriptional changes.

It should also be noted that a common problem in studies where plant growth is affected, is separating primary and secondary effects. That is, determining which, if any, of the transcriptional changes cause the growth phenotype and which are a consequence of the growth phenotype. Here we present all the significant transcript changes, to serve as a starting point for further research. Tracking transcriptional changes over time may help to distinguish between primary and secondary transcriptional effects, whereby transcripts affected very early in development may be causative but those that only appear later may be secondary effects.

## 4 Conclusions

In this report, we describe the detailed transcriptional profile of plants with genetically enhanced AOX1A, and of plant lines with modified NDB2 expression. A wide range of transcriptional changes were observed as a consequence of this genetic manipulation, even though plants were grown under non-limiting conditions. These include changes in the expression of:

1. Photosynthesis genes, with PSII reaction centre genes down-regulated in response to increased AOX1A:NDB2 and PSI and PSII light harvesting genes down-regulated in all lines with increased AOX1A transcript. Typically, a loss of AOX1 limits the efficiency of photosynthesis, but according to these transcriptional changes, increasing AOX1A expression by itself may also limit photosynthetic capacity.
2. Sugar signaling and transport genes e.g. DIN10, ASN3 and MIOX2 and SWEET1,2,1. These changes suggest increased mobilization of carbohydrate stores in lines overexpressing AOX1A.
3. Hormone-related genes including ethylene biosynthesis genes and response factors, small auxin upregulated RNAs, down-regulation of abscisic acid biosynthesis genes and up-regulation of abscisic acid catabolism genes, jasmonate biosynthesis genes, down-regulation of salicylic acid biosynthesis and responsive genes, and up-regulation of gibberellin biosynthesis genes but down-regulation of GA4 biosynthesis genes (A form of gibberellin). These implicate abscisic acid, ethylene, salicylic acid and gibberellins in AOX-regulated growth.
4. Secondary metabolism genes coordinating flavonoid, phenylpropanoid, lignin and glucosinolate metabolism
5. Abiotic and biotic stress-responsive genes, including defensins, heat shock protein genes, redox-responsive transcription factors, DREBs and cell wall related genes (potentially promoting cell wall degradation).
6. A suite of transcription factors, most notably WRKY40 and other WRKY TFs (e.g. WRKY8, 27 and 30).

Based on the above, the following analyses of these plants may be useful:

1. Gas exchange and chlorophyll fluorescence measurements under standard growth conditions, particularly looking at rates of photosynthesis and PSII efficiency.
2. Quantification of sugars and other metabolites.
3. Endogenous concentrations of hormones, and the effect of ethylene, abscisic acid, salicylic acid or gibberellin (especially GH4) treatments on the delayed growth phenotype.
4. Assessment of secondary metabolites using a metabolomics approach, or targeted towards phenylpropanoids and flavonoids.
5. Tolerance to additional stress treatments including starvation (i.e. extended darkness or low CO_2_) or pathogen attack.
6. Measurement of transcriptional effects during stress exposure.

Based on the above findings, it is surprising that growth was not more affected in the AOX1A-OEX and *ndb2* lines under normal conditions (Sweetman et al., 2019). However, in this preliminary study we have focused on transcript levels, and these do not always correlate with protein abundance, nor enzyme activity, due to post-transcriptional and post-translational regulation. Further investigations of our various lines are required to determine how proteins and activities are affected by changes in AOX1A and NDB2 protein levels. Further studies with purified mitochondria from these lines together with oxygen discrimination and flux analyses are also necessary to determine whether there are any changes to mitochondrial activities *in vivo*.

AOX1A has been linked to retrograde signaling processes throughout plant cells (Van Aken et al., 2009). Our study confirms this, showing that expression of genes encoded not only in the mitochondria and nucleus, but the chloroplast as well, are changed when AOX expression is altered. For example, signaling processes that lead to down-regulation of chloroplast-encoded transcripts appear to be dependent on the relative abundance of AOX1A and NDB2. Over-expressing NDB2 together with AOX1A appears to reverse the general down-regulation of photosynthesis-related transcripts in response to increased AOX1A alone and this may account for the delayed growth phenotype seen in the latter line but not the former (Sweetman et al., 2019). It is well known that alternative respiration in the mitochondrion is fundamentally linked to photosynthetic performance, especially under stress conditions.

Overall, this report demonstrates that physiological effects of altered mitochondrial electron transport might not be due directly to the changes in activity of AOX1A and NDB2 *per se*. Rather, a change in AOX1A:NDB2 might initiate signalling processes that impact other compartments of the cell and consequential transcriptional changes might affect plant growth. While such signalling responses have been seen in other lines with decreased AOX1A expression (e.g. Giraud et al., 2008), this is the first study to look at effects of altering expression of both AOX1A and NDB2. Importantly, we have identified potential mechanisms for the observed physiological effects, which now need to be experimentally tested. Future work will elucidate the metabolic effect of disrupting the AOX1A:NDB2 balance and an investigation of the transcriptomic and metabolomic responses when these lines are exposed to a stress.

## Dedication

This manuscript is dedicated to the memory of Jim Siedow. An inspirational mentor, colleague and friend. KLS.

## Data availability statement

The data presented in the study are deposited in the Sequence Read Archive (NCBI) repository, accession number PRJNA896774.

## Author contributions

KS and CJ conceived the project. CW carried out all experimental procedures. DW carried out all data processing. CS interpreted the data and drafted the manuscript with feedback from DD, KS and CJ. All authors contributed to the article and approved the submitted version.

## Funding

This work was funded by The Australian Research Council, Discovery Project DP140103090 and a Flinders University Internal Grant, Project 10683.

## Acknowledgments

RNA sequencing services were provided by Flinders Genomics Facility, Adelaide, Australia

## Conflict of interest

The authors declare that the research was conducted in the absence of any commercial or financial relationships that could be construed as a potential conflict of interest.

## Publisher’s note

All claims expressed in this article are solely those of the authors and do not necessarily represent those of their affiliated organizations, or those of the publisher, the editors and the reviewers. Any product that may be evaluated in this article, or claim that may be made by its manufacturer, is not guaranteed or endorsed by the publisher.
