# Supplementary material for: Altering the balance between AOX1A and NDB2 expression affects a common set of transcripts in Arabidopsis
Source: Front Plant Sci. 2022 Nov 15;13:876843. doi: 10.3389/fpls.2022.876843 (PMC9716356; doi:10.3389/fpls.2022.876843)
Supplement: Supplementary file 1 [file DataSheet_1.pdf]

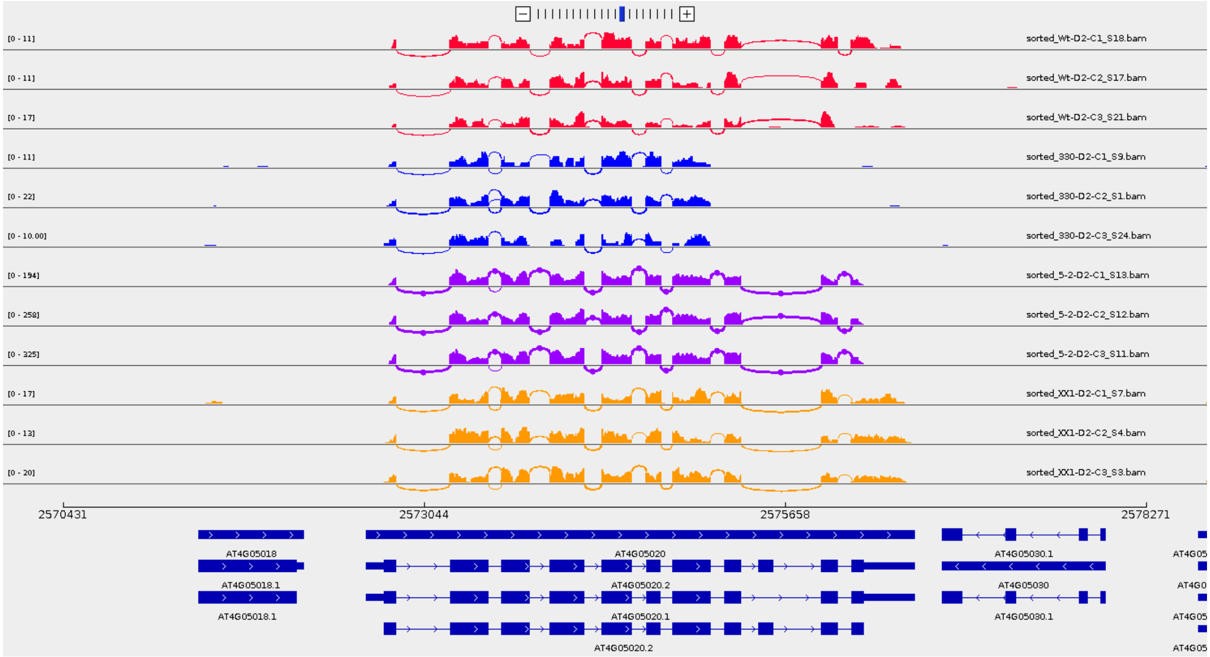

Figure S1: Mapped reads at NDB2 indicating truncated NDB2 transcript in ndb2 knockout line. This sashimi plot depicts the coverage of each alignment track, i.e. wild type (red), *ndb2* (blue), dual-OEX (purple) and AOX1A-OEX (yellow), plotted as a bar graph on the NDB2 gene. The gene structure (including exons, introns and junctions) and genomic coordinates of functional *A. thaliana* NDB2 on Chr 4 is depicted in blue at the bottom panel. NDB2 transcripts of the ndb2 knockout line are truncated at exon 7. NDB2 transcripts of the dual-OEX line are missing part of exon 10/11, as the cloned gene within the overexpression cassette terminates shortly after the stop codon (i.e. lacks 3'UTR).

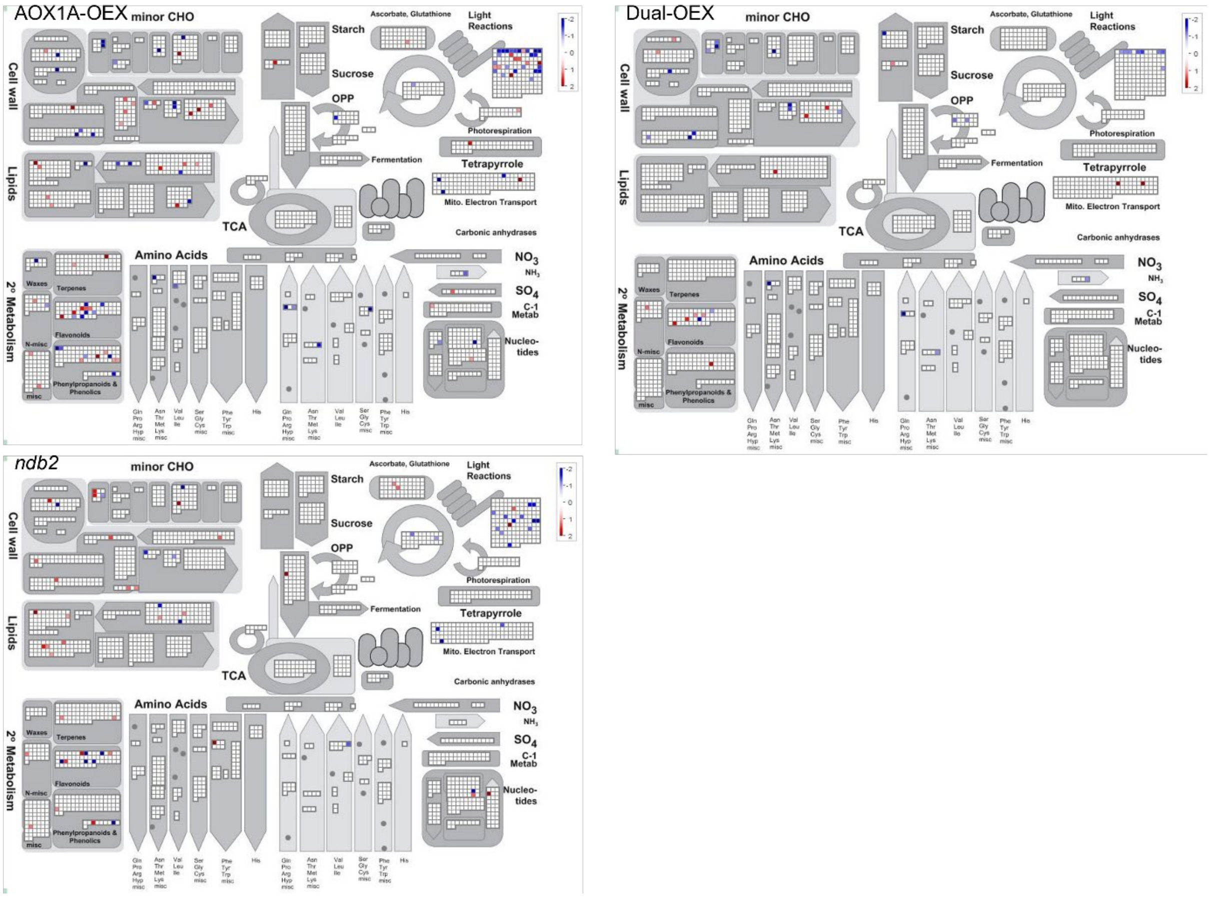

Figure S2: Overview of DEGs relevant to the major areas of metabolism, using the MapMan Metabolism Overview pathway. The key shows an increase (red) or decrease (blue) of transcript levels based on DEGs with $\log_2$ >1 or <-1 and an FDR-adjusted p-value < 0.05.

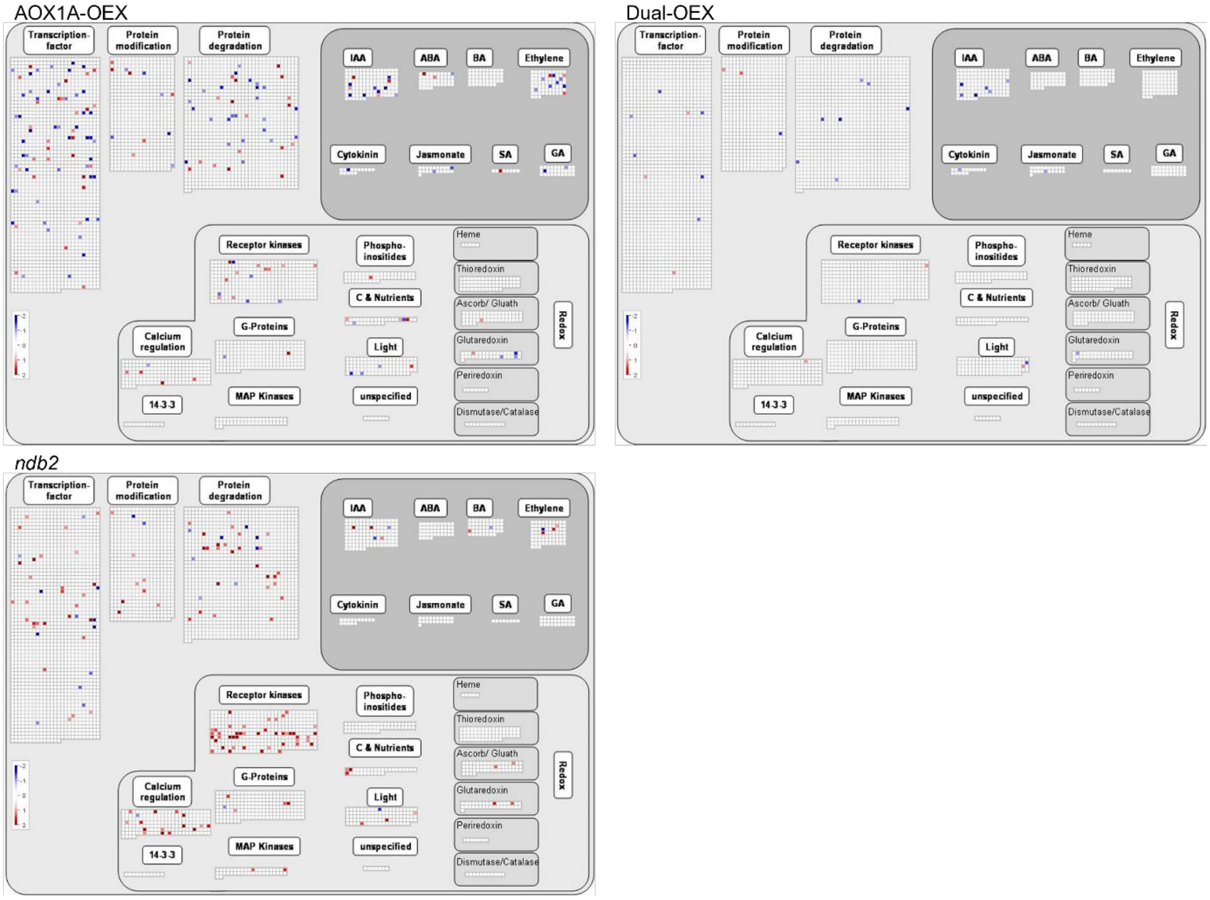

Figure S3: Overview of DEGs relevant to the major areas of cellular regulation, using the MapMan Regulation Overview pathway. The key shows an increase (red) or decrease (blue) of transcript levels based on DEGs with log_2 >1 or <-1 and an FDR-adjusted p-value < 0.05.

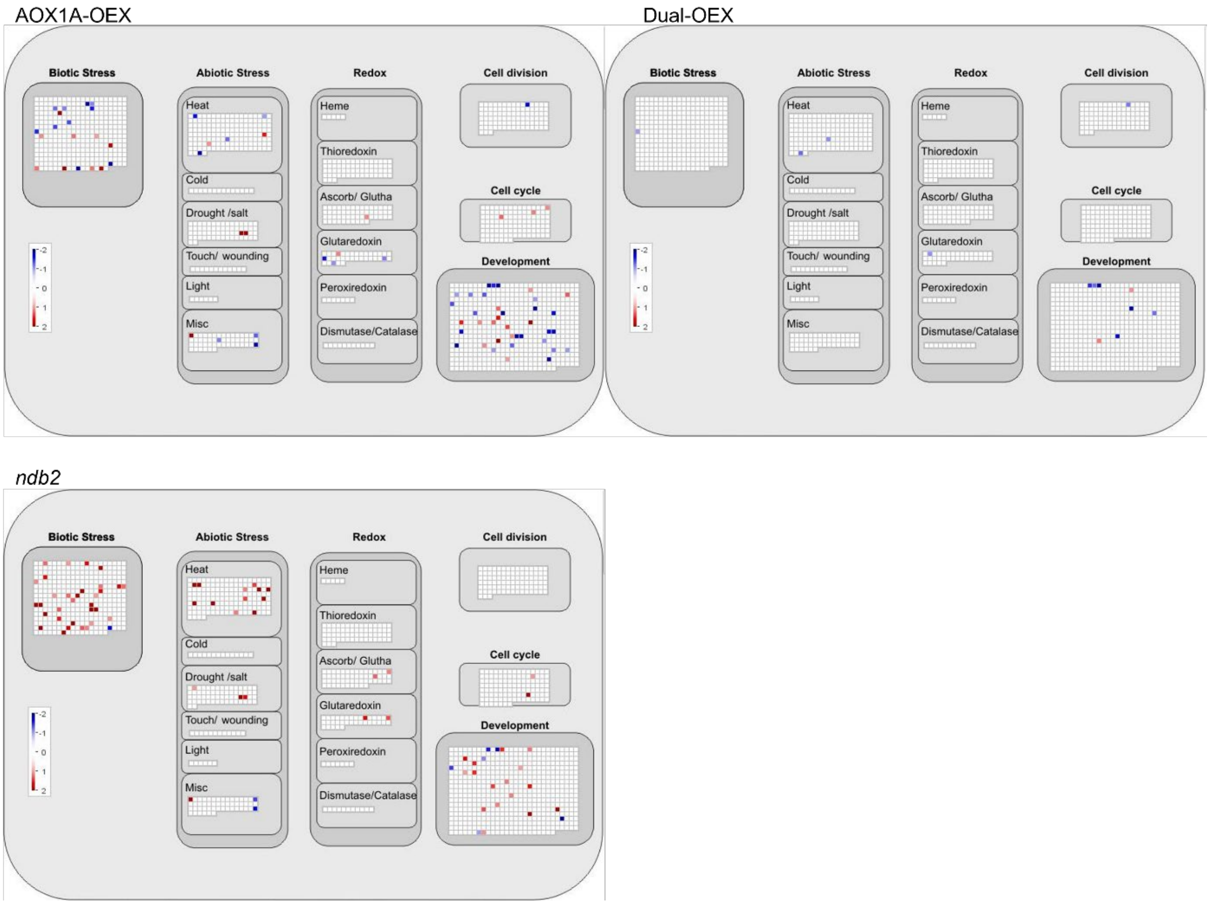

Figure S4: Overview of DEGs relevant to different types of cellular responses, using the MapMan Cellular Response Overview pathway. The key shows an increase (red) or decrease (blue) of transcript levels based on DEGs with log_2 >1 or <-1 and an FDR-adjusted p-value < 0.05.

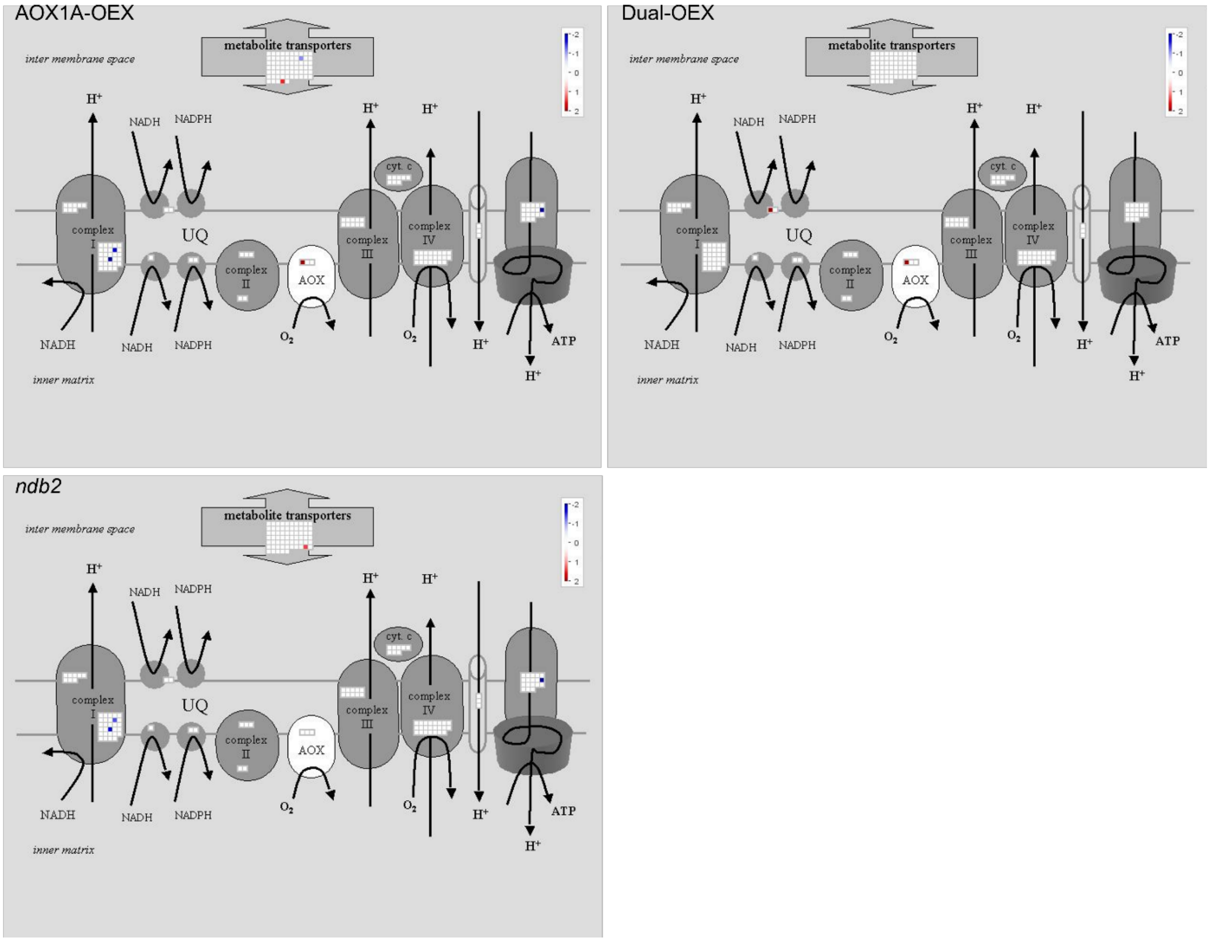

Figure S5: Overview of DEGs relevant to the mitochondrial electron transport chain, using the MapMan Mitochondrial e-Transport pathway. The key shows an increase (red) or decrease (blue) of transcript levels based on DEGs with log_2 >1 or <-1 and an FDR-adjusted p-value < 0.05. Gene details and transcript data can be found in Tables S5 and S6.

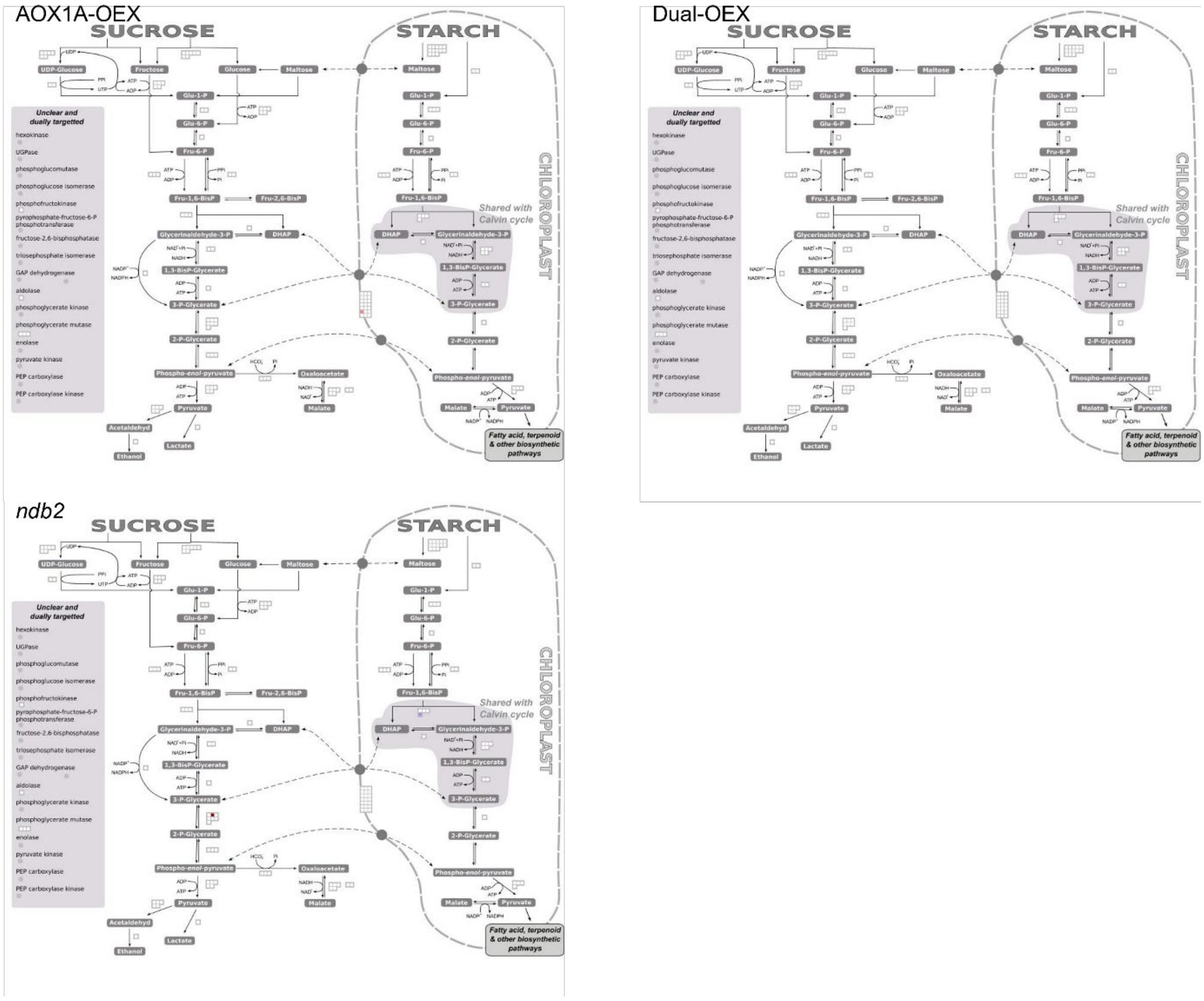

Figure S6: Overview of DEGs relevant to glycolysis and carbohydrate metabolism, using the MapMan Plant Glycolysis pathway. The key shows an increase (red) or decrease (blue) of transcript levels based on DEGs with $\log_2$ >1 or <-1 and an FDR-adjusted p-value < 0.05. Gene details and transcript data can be found in Table S7

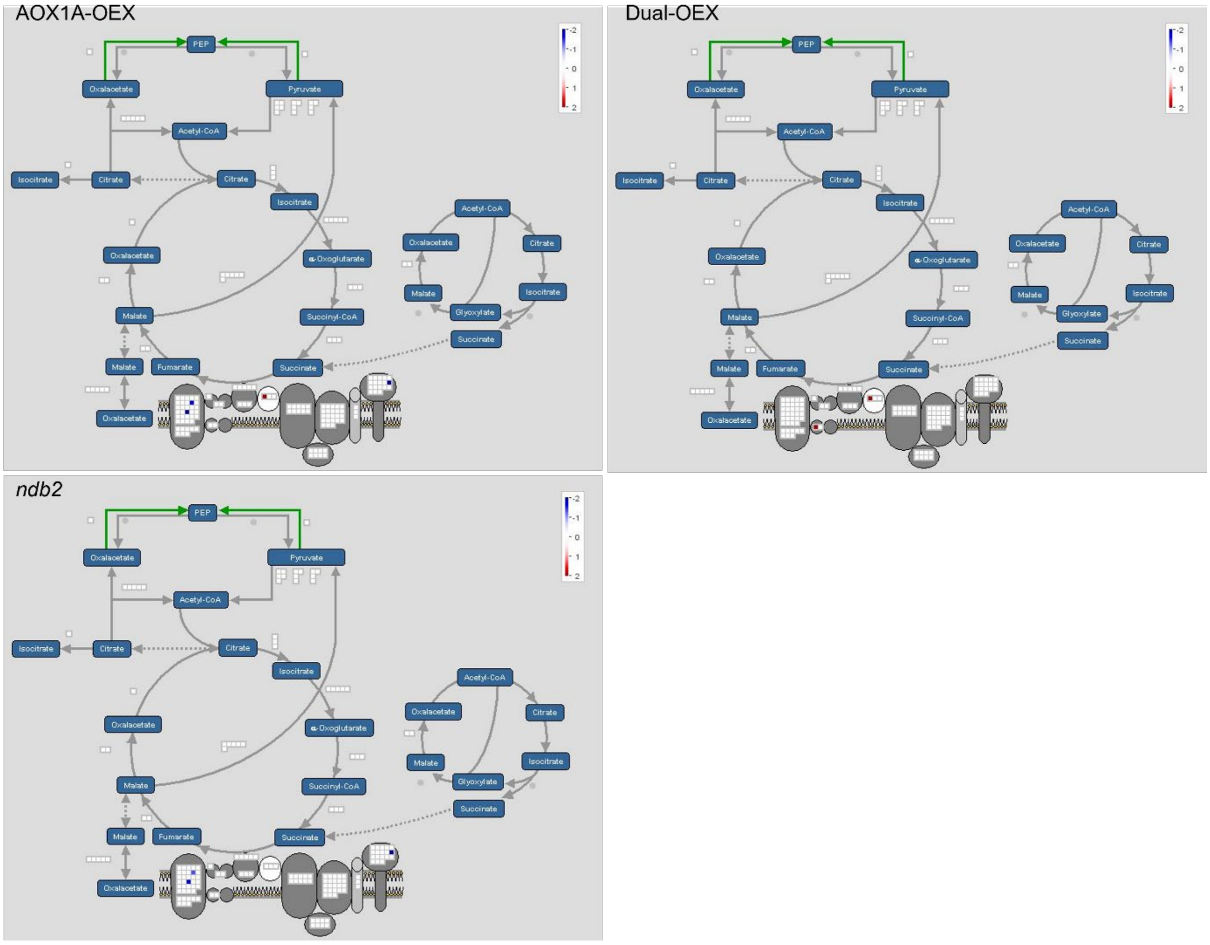

Figure S7: Overview of DEGs relevant to the TCA cycle, using the MapMan TCA pathway. The key shows an increase (red) or decrease (blue) of transcript levels based on DEGs with log_2 >1 or <-1 and an FDR-adjusted p-value < 0.05. Gene details and transcript data can be found in Table S7.

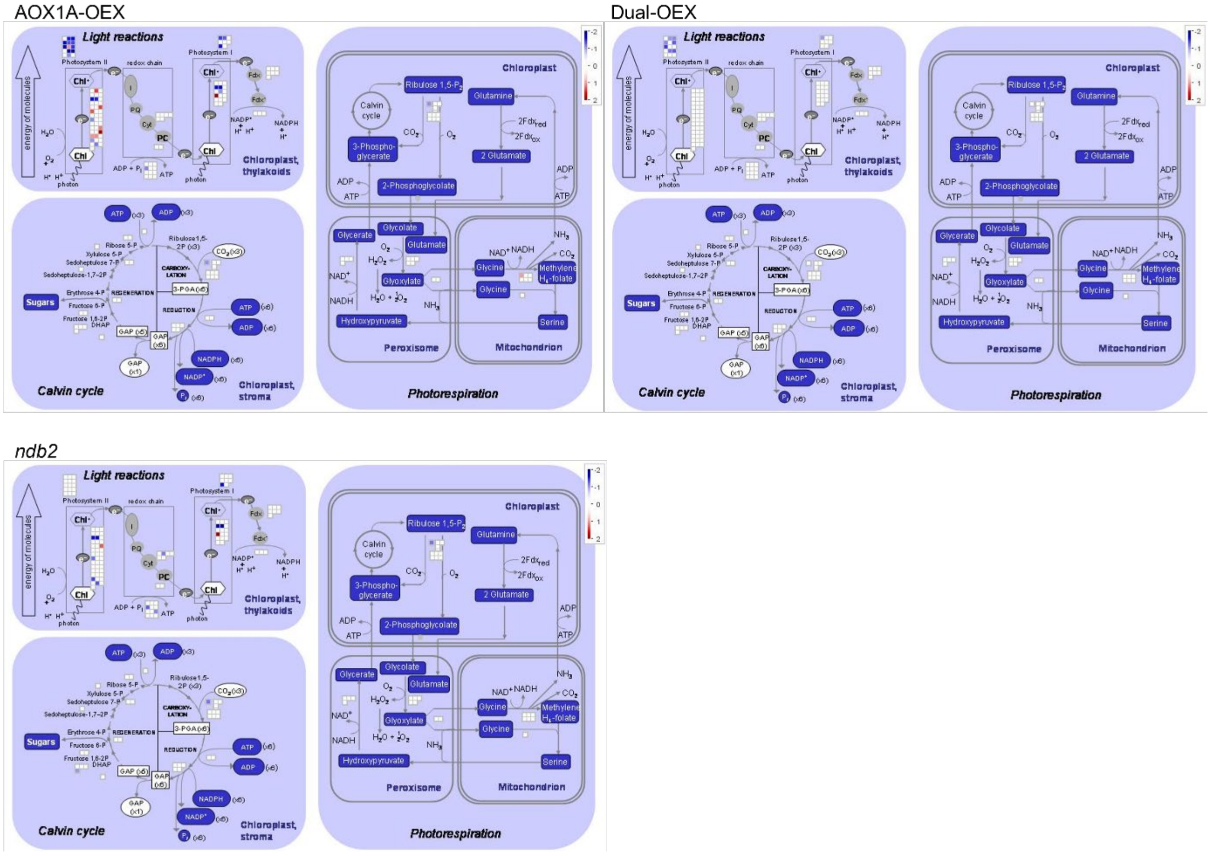

Figure S8: Overview of DEGs involved in photosynthesis, using the MapMan Photosynthesis pathway. The key shows an increase (red) or decrease (blue) of transcript levels based on DEGs with log_2 >1 or <-1 and an FDR-adjusted p-value < 0.05. Gene details and transcript data can be found in Table S8.

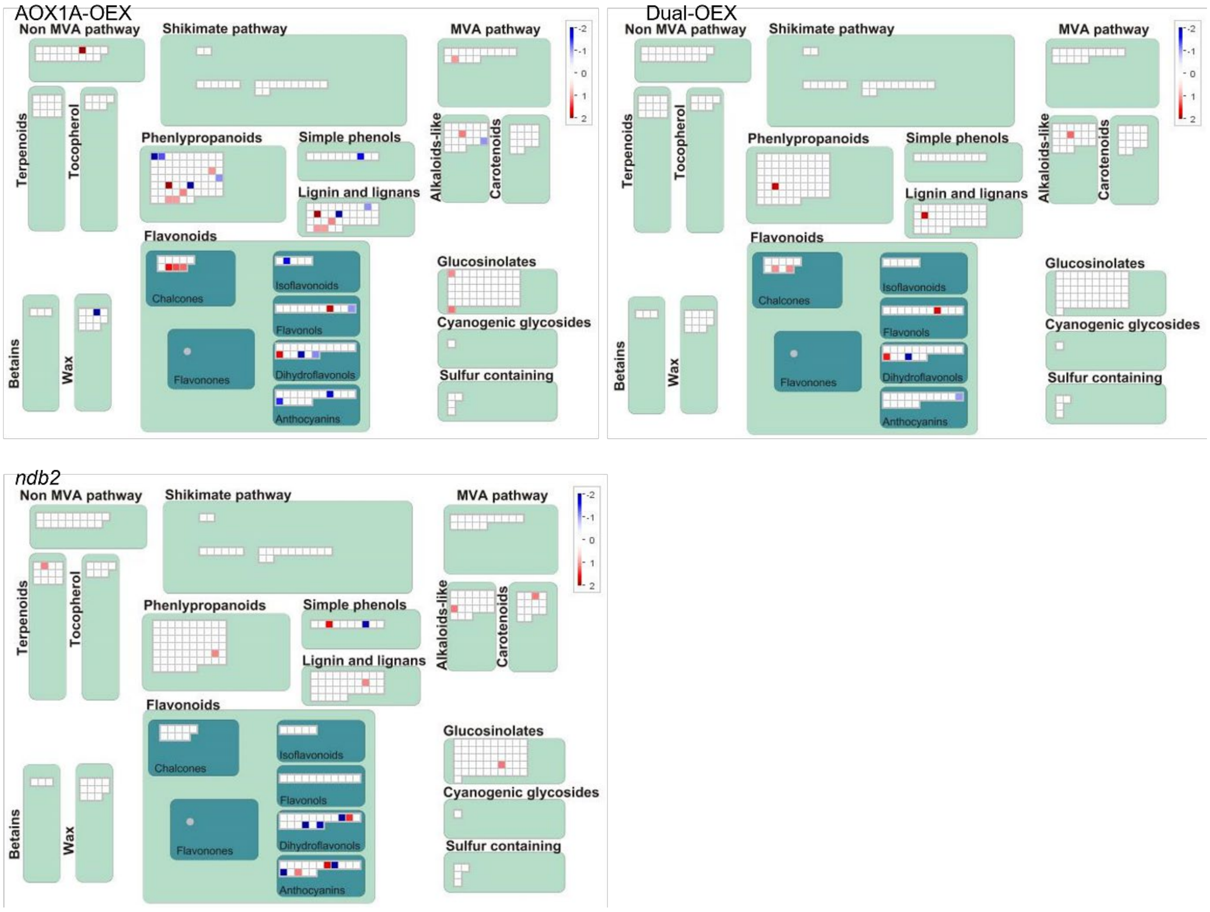

Figure S9: Overview of DEGs relevant to various pathways of secondary metabolism, using the MapMan Secondary Metabolism pathway. The key shows an increase (red) or decrease (blue) of transcript levels based on DEGs with $\log_2$ >1 or <-1 and an FDR-adjusted p-value < 0.05.

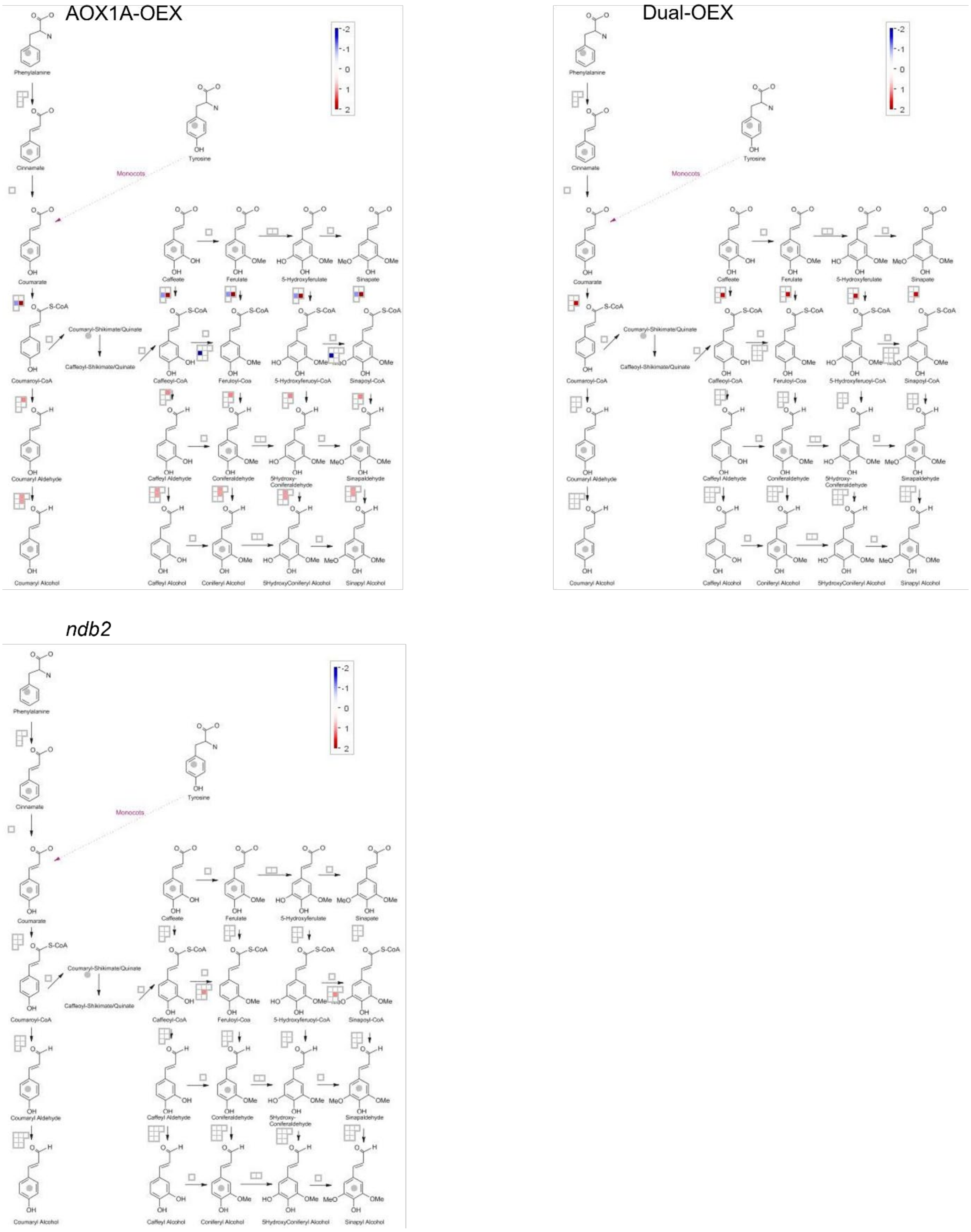

Figure S10: Overview of DEGs involved in biosynthesis of phenylpropanoids, using the MapMan Phenylpropanoid Biosynthesis pathway. The key shows an increase (red) or decrease (blue) of transcript levels based on DEGs with $\log_2$ >1 or <-1 and an FDR-adjusted p-value < 0.05.

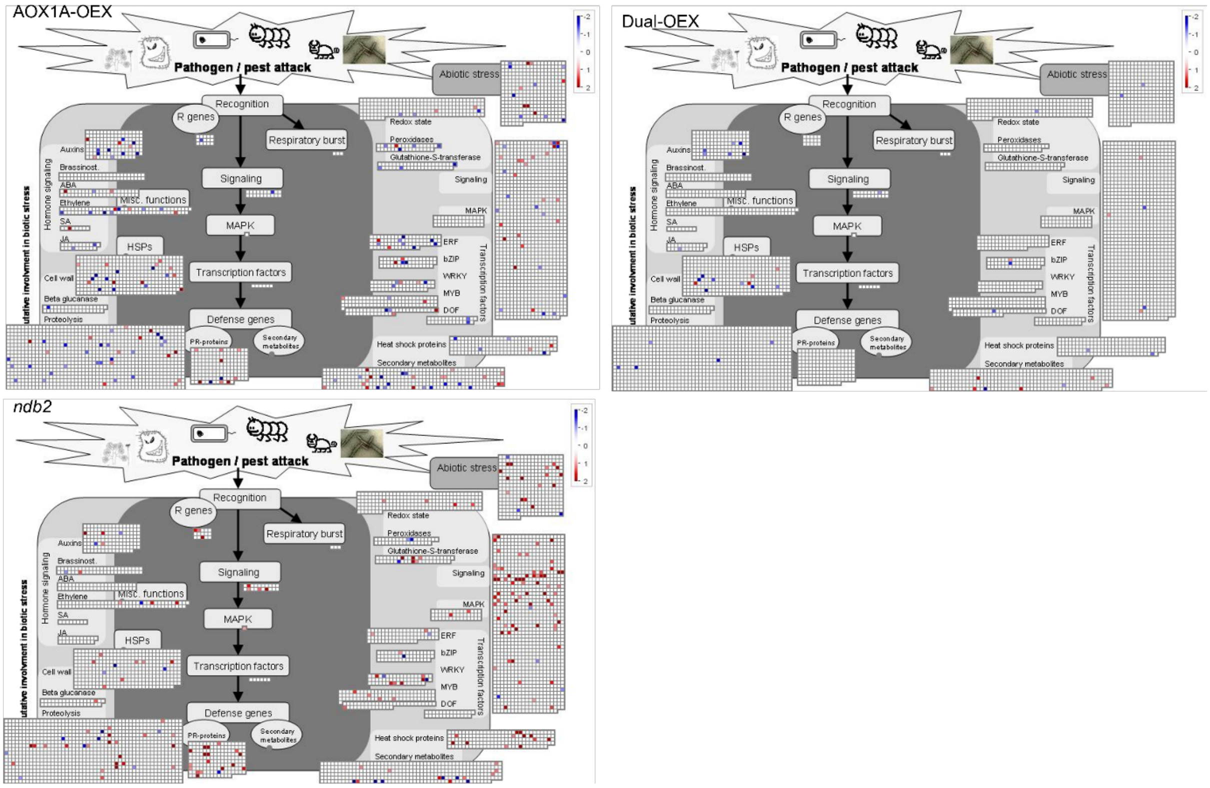

Figure S11: Overview of DEGs involved in biotic and abiotic stress, using the MapMan Biotic Stress pathway. The key shows an increase (red) or decrease (blue) of transcript levels based on DEGs with $\log_2 > 1$ or < -1 and an FDR-adjusted p-value < 0.05.

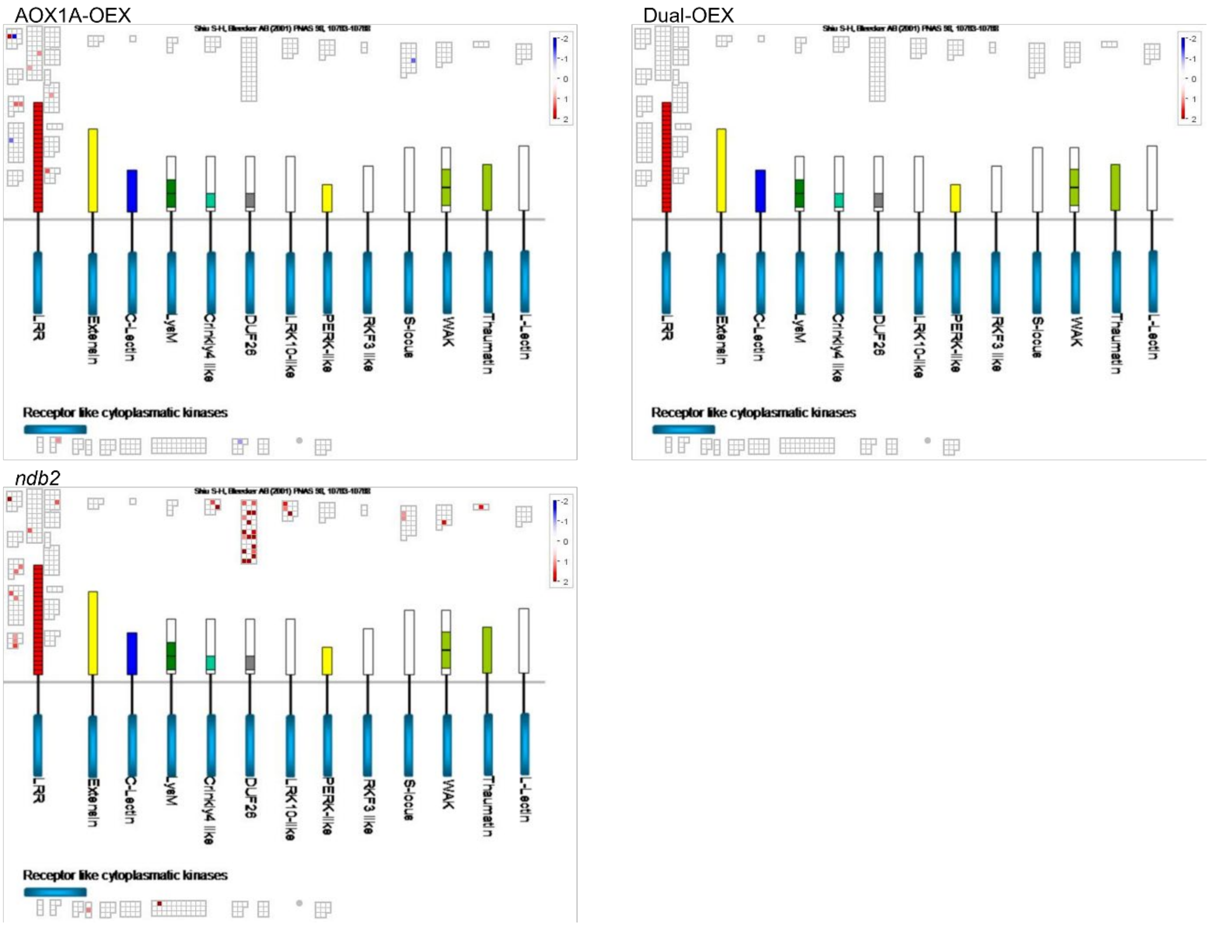

Figure S12: Overview of DEGs potentially involved receptor kinase activity, using the MapMan Receptor Like Kinase pathway. The key shows an increase (red) or decrease (blue) of transcript levels based on DEGs with log_2 >1 or <-1 and an FDR-adjusted p-value < 0.05.

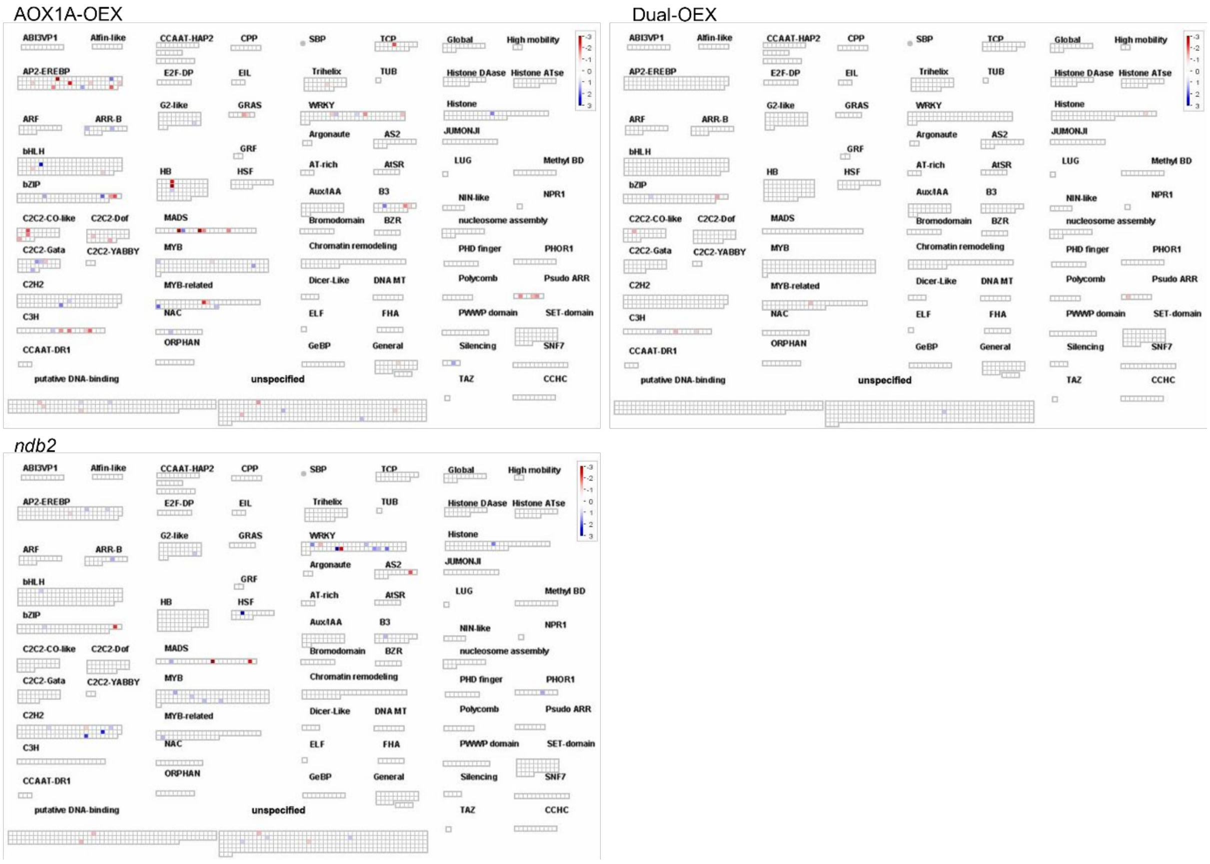

Figure S13: MapMan Transcription Factors Overview of DEGs potentially involved in transcriptional regulation, using the MapMan Transcription pathway. The key shows an increase (red) or decrease (blue) of transcript levels based on DEGs with $\log_2 > 1$ or < -1 and an FDR-adjusted p-value < 0.05.
